# Pragmatists, Positive Communicators, and Shy Enthusiasts: Three Viewpoints on Web Conferencing in Health Sciences Education

**DOI:** 10.2196/jmir.9.5.e39

**Published:** 2007-12-31

**Authors:** Ruta Valaitis, Noori Akhtar-Danesh, Kevin Eva, Anthony Levinson, Bruce Wainman

**Affiliations:** Faculty of Health SciencesMcMaster UniversityHamilton, ONCanada

**Keywords:** Web conferencing, Q-methodology, synchronous communication, e-learning, distance education, Internet

## Abstract

**Background:**

Web conferencing is a synchronous technology that allows coordinated online audio and visual interactions with learners logged in to a central server. Recently, its use has grown rapidly in academia, while research on its use has not kept up. Conferencing systems typically facilitate communication and support for multiple presenters in different locations. A paucity of research has evaluated synchronous Web conferencing in health sciences education.

**Objective:**

McMaster University Faculty of Health Sciences trialed Wimba’s Live Classroom Web conferencing technology to support education and curriculum activities with students and faculty. The purpose of this study was to explore faculty, staff, and student perceptions of Web conferencing as a support for teaching and learning in health sciences. The Live Classroom technology provided features including real-time VoIP audio, an interactive whiteboard, text chat, PowerPoint slide sharing, application sharing, and archiving of live conferences to support student education and curriculum activities.

**Methods:**

Q-methodology was used to identify unique and common viewpoints of participants who had exposure to Web conferencing to support educational applications during the trial evaluation period. This methodology is particularly useful for research on human perceptions and interpersonal relationships to identify groups of participants with different perceptions. It mixes qualitative and quantitative methods. In a Q-methodology study, the goal is to uncover different patterns of thought rather than their numerical distribution among the larger population.

**Results:**

A total of 36 people participated in the study, including medical residents (14), nursing graduate students (11), health sciences faculty (9), and health sciences staff (2). Three unique viewpoints were identified: pragmatists (factor 1), positive communicators (factor 2A), and shy enthusiasts (factor 2B). These factors explained 28% (factor 1) and 11% (factor 2) of the total variance, respectively. The majority of respondents were pragmatists (n = 26), who endorsed the value of Web conferencing yet identified that technical and ease-of-use problems could jeopardize its use. Positive communicators (N = 4) enjoyed technology and felt that Web conferencing could facilitate communication in a variety of contexts. Shy enthusiasts (N = 4) were also positive and comfortable with the technology but differed in that they preferred communicating from a distance rather than face-to-face. Common viewpoints were held by all groups: they found Web conferencing to be superior to audio conferencing alone, felt more training would be useful, and had no concerns that Web conferencing would hamper their interactivity with remote participants or that students accustomed to face-to-face learning would not enjoy Web conferencing.

**Conclusions:**

Overall, all participants, including pragmatists who were more cautious about the technology, viewed Web conferencing as an enabler, especially when face-to-face meetings were not possible. Adequate technical support and training need to be provided for successful ongoing implementation of Web conferencing.

## Introduction

### Synchronous Conferencing Technologies and e-Learning

There has been increasing investment in the application of e-learning technologies in Canadian educational institutions. Yet a recent review of e-learning research in Canada indicated that the majority of the research appears to have focused on distance education, with less attention placed on hybrid/blended (mixed online and face-to-face) learning contexts. Findings from their review of post secondary education research showed that the appropriate use of computer-mediated education can enrich the learning environment, reduce isolation, and increase motivation for distance learners [1].

Due to the expansion of multi-site and collaborative undergraduate and graduate programs, the struggle to meet the needs of students who are juggling work and school, and the growing demands for an increased health professional workforce in Canada [2], health sciences education programs are challenged to find effective ways to reach learners in real time. Web conferencing trends continue to escalate to support collaborative work in industry [3] as well as education [4,5]. Educational applications have moved beyond classroom work to include support for administrative meetings, interviews, and even Web casts of commencement ceremonies [6]. As applications of synchronous (real-time) technologies to support colleges and universities have grown, the research on their use has not kept up. Although much has been written about the use of e-learning to support health sciences education, a review of e-learning practices in undergraduate medical education found few reported studies on the use of synchronous communication technologies [7].

### Videoconferencing Versus Web Conferencing

Videoconferencing and Web conferencing are both synchronous communication technologies. A videoconferencing system allows people in different locations to interact via video and audio, most frequently with dedicated video and telephone equipment set up in a special-purpose room—often due to the requirement of special cameras, microphones, and dedicated telephone lines (eg, T1 or ISDN). Thus, videoconferencing often requires that participants travel to designated conferencing sites to connect to other remote sites. In contrast with older videoconferencing systems’ dependence on analog signals and telephone equipment, Web conferencing enables collaborative interaction using voice over Internet protocol (VoIP) communication between a network of computers, which can share images, presentations, and computer applications, connecting from desktop computers in remote locations [6]. Many Web conferencing systems support the streaming of video as well as audio and presentation images. Improvements in streaming technologies and bandwidth have contributed to the growth of Web conferencing.

### Synchronous Conferencing Technologies in Health Sciences Education

Such synchronous conferencing technologies have been used to support health sciences education; however, most evaluations report their use in continuing education and graduate programs. Gagliardi et al [8] conducted a pilot study in which community-based surgeons used videoconferencing to support multidisciplinary oncology rounds. Participants completed surveys which showed that they generally felt positive about the videoconferencing, and the authors concluded that it is possible to engage participants remotely through videoconferencing. Odell et al [9] assessed the feasibility of using videoconferencing to support dental postgraduate education in the United Kingdom. Teachers completed surveys immediately after conferences and participated in a follow-up interview 1 week after each conferencing session; 27 teachers involved in 41 sessions were included in the sample. Most teachers preferred videoconferencing to on-site teaching due to savings in travel time; however, they also experienced a sense of distance with the audience and had difficulties managing question and answer periods. Training was felt to be essential for successful videoconferencing.

Locatis and colleagues [10] evaluated a mix of videoconferencing, Web casting, and chat technology delivered over the Internet to engage health professionals in a conference. They found that although it was technically feasible to deliver a live Web cast with additional chat facilities, difficulties with latency of audio and visual displays were problematic. Up to 2-minute delays created significant synchronous communication problems, which were felt to be related to differences in streaming formats. Participants were also unfamiliar with viewing a table of images from multiple sites compared to a typical single image broadcast. Connection problems were also experienced due to issues with firewalls, bandwidth limitations, and local network configurations.

We found a general paucity of research that evaluated Web conferencing in health sciences education. The research was generally found in white papers, and peer-reviewed papers were limited to reports of experiences and/or results from satisfaction surveys. An evaluation of Web conferencing by public health professionals who participated in monthly development sessions on emergency preparedness indicated that technical problems decreased quickly after a single experience with the technology [11]. Web casting using Mediasite (Sonic Foundry, Inc) was evaluated as a tool to support a graduate nursing program [12]. Mediasite can broadcast video streams of the lecturer as well as push presentation images from the presenter’s computer. Polls, question-and-answer, and text chat features were used to promote communication. Students’ evaluations (N = 27) indicated that most connected from home and used broadband access. The quality of the broadcast diminished with dial-up connections. Overall, participants were satisfied with the technology and their interaction with the instructor and appreciated the cost savings resulting from decreased travel time. However, some participants missed the human interaction while online. Ostrow and DiMaria-Ghalili [13] reported on 20 years’ of experience in delivering graduate nursing education by distance, which most recently included Web casting. They described lessons learned from their experiences, including the need for a solid orientation to the technology, reliable easy-to-use technology with technical support available 24/7, the provision of prompt and highly responsive feedback to students, and institutional partnerships to support students. They noted that mature students who started the program with little technical savvy left with highly improved technical skills. They felt that the use of technology increased student recruitment and enrollment and program cost-effectiveness.

In addition to supporting health sciences education, Web conferencing has been used with success to support a health sciences research “collaboratory” [14]. The collaboratory involved an oral cancer center and an HIV/AIDS center in the United States; virtual meetings were supported using Web conferencing technologies (NetMeeting, Placeware). The virtual meetings aided communication on the initiation of joint studies and data analysis. Over time, larger group meetings became less frequent and more one-on-one, cross-site meetings occurred between researchers. Well-attended webinars (PlaceWare and conference calls) were used to broadcast presentations of pre-publication data among involved research centers.

**Figure 1:**
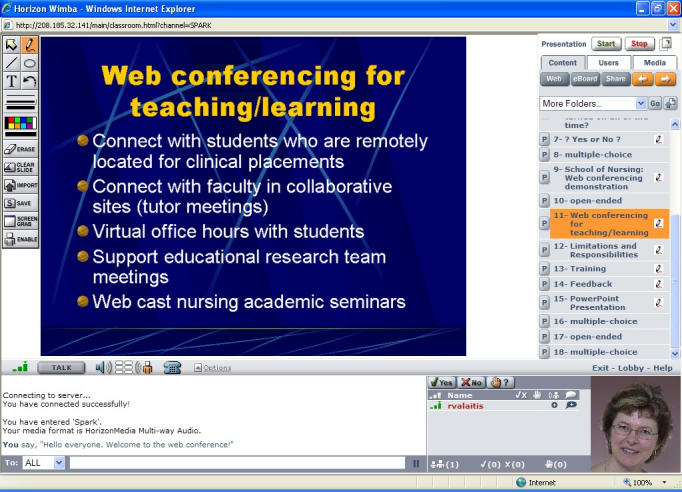
Screen capture of Web conferencing interface of Live Classroom

In 2005, McMaster’s Faculty of Health Sciences trialed the use of Web conferencing technology with students, administrative staff, and faculty of undergraduate and graduate programs in medicine, midwifery, nursing, and rehabilitation sciences. Wimba Inc’s Live Classroom technology was used, which provided features including real-time VoIP audio, an interactive whiteboard, text chat, PowerPoint slide sharing, application sharing, and archiving of live conferences to support student education and curriculum activities (Figure 1).

It was timely to examine student, staff, and faculty views on the use of Web conferencing since use of this technology has continued to grow [6] and has the potential to address problems in evermore complex and expanding health sciences education programs. It is critical to explore instructor, staff, and learner viewpoints during the initial application of a learning innovation to inform uptake and acceptability of the technology. The purpose of this study was, therefore, to investigate faculty, student, and support staff perceptions of the use of Web conferencing as a support for teaching and learning in health sciences using Q-methodology. This paper also demonstrates the unique contribution of the use of Q-methodology in health sciences educational research, which capitalizes on the benefits of qualitative and quantitative approaches.

## Methods

### Sampling and Recruitment

Faculty, staff, and students in the Faculty of Health Sciences who participated in one or more Web conferences from August 2005 to January 2006 were asked to take part in the evaluation. During this time, Web conferencing was used to support weekly graduate nursing seminars, academic rounds for medical residents, and faculty meetings with faculty located at multiple sites. The first author oversaw the administration of Web conferencing technology in the Faculty of Health Sciences during this phase. Faculty who booked use of the technology over this time frame were invited to participate in the study. All faculty and staff who had booked a Web conference during the trial period were asked to forward an email invitation for the study to their students and faculty who had participated in a Web conference. A nonrandom convenience sample of participants was approached, including (1) an anesthesiologist and his residents; (2) graduate students in nursing, graduate nursing faculty, administrative staff, and guest presenters who were involved in weekly graduate seminars; and (3) faculty members of the Nursing Information and Communication Technology Committee who taught in the undergraduate nursing program. Groups who attended a data collection meeting to complete the Q-sort exercise received refreshments; in addition, every participant also received a Can $5 coffee shop gift certificate. Ethics approval was received for the study from the McMaster University Research Ethics Board.

### Q-Methodology

Q-Methodology was used to identify common viewpoints of students, faculty, and administrative staff who had exposure to Web conferencing. This method has been used in different aspects of health sciences research, including evaluation of job satisfaction [15], patients’ viewpoints on health and rehabilitation [16], use of research information in clinical decision making [17-19], and exploration of nursing attitudes toward health promotion [20]. Many educational studies seek to understand satisfaction and perceived usefulness of different educational strategies by educators and students, hence our desire to also employ this method and receive feedback on its feasibility and effectiveness.

Q-methodology was introduced in 1935 by Stephenson [21,22] and was only employed sporadically until recently emerging as a more widely used method, mainly because of advances in the statistical analysis component [22]. This method is used to identify unique viewpoints as well as commonly shared views, and it is particularly valuable in research that explores human perceptions and interpersonal relationships [15]. The method allows the researcher to identify groups of participants having similar and alternate viewpoints, and, in turn, to ascertain similarities and differences between groups. It mixes qualitative and quantitative methods. In a Q-methodology study, the goal is to uncover different patterns of thought rather than their numerical distribution among the larger population. In other words, the number of participants is not the important issue; rather, it is the representation of different points of view about the topic of study [23]. Q-studies typically use small sample sizes compared to, for example, survey research, and low response rates do not bias the results because the primary objective is to identify a typology, not to test the typology’s proportional distribution within the larger population [24]. Brown [25] recommends that 40-60 participants are more than adequate for most studies, and far fewer may be needed for some specific studies. He maintains that “what is of interest ultimately are the factors with at least four or five persons defining each; beyond that, additional subjects add very little.” Therefore, a factor with at least four subjects and an eigenvalue greater than one would be considered a significant factor. In this study, we approached approximately 50 individuals who had exposure to Web conferencing during the trial period.

Q-methodology uses correlation and by-person factor analysis (ie, the statistical analysis is performed by person rather than by variable, trait, or statement). Respondents are grouped based on the similarities of their Q-sorts, with each group (or factor) representing individuals with similar views, feelings, or experiences about the topic. Each individual with a significant loading (*P* < .05) on one factor is counted on that factor. A factor loading is a correlation between a Q-sort and the factor itself. The standard error of this correlation is estimated by, *SE* = 1/√N where N is the number of statements [24]. Then, a correlation is statistically significant if it is about 2 to 2.5 times the standard error.

In other contexts, the test-retest reliability of Q-sorting has been found to be 0.80 or higher [26,27]. Content validity is typically assessed by literature review and a team of 3-5 domain experts and is tested in one or more pilot studies. The face validity of the statements is assured by using participants’ exact wording of the statements with slight editing only for grammar and readability [28]. Member checking (ie, reviewing factor interpretation with participants) is also useful but could not be included in this study because data collection and analyses were completed after many students had finished their programs and we had not received ethics clearance to track participants.

Positive, negative, and neutral statements about the use of Web conferencing technology were collected during an earlier evaluation of Live Classroom (Wimba, Inc, New York, NY, USA) in the Faculty of Health Sciences. Comments were gathered from responses to an open-ended question in an online evaluation; we also invited Web conferencing users to share their thoughts about Web conferencing. Specifically, we asked them to email at least five statements that reflected how they felt about Web conferencing based on their experience with Live Classroom. They were instructed that “statements should indicate strengths, limitations, barriers or any other things that you think are important for us to know about the technology.”Over 100 statements were compiled into one dataset (the concourse). To have a representative Q-sample, we used an inductive process as there was no theoretical hypothesis or framework involved. The statements in the concourse were classified into six domains emerging from the statements themselves, including teaching and learning, access/reach, communication, technical features, technology setup and training, and comfort / ease of use with technology. The statements within each domain were refined, clarified, and significantly reduced by the research team. An iterative consensus process was engaged in which each coauthor independently considered how the statements might be combined, rephrased, or deleted for the sake of clarity and avoidance of redundancy. This process was followed by a group meeting and then more independent consideration, continuing back and forth in this way until consensus had been achieved regarding the most appropriate list of statements. The final set included 42 statements (Multimedia Appendix 1) that represented key ideas from each domain about the use of Web conferencing in education.

Four volunteers agreed to pilot-test the tool, which resulted in minor edits to clarify some statements. Invited participants were then asked to sort the randomly numbered final statements onto a grid, scoring each statement between −4 and +4, where negative scores indicated disagreement, until all blanks on the grid were completed. The grid was constructed such that participants could only assign two statements a score of −4 and two statements a score of +4. Three statements could get a score of −3 and three could score +3, and so on. Detailed instructions, including an example, were provided to participants (Multimedia Appendix 2). The Q-sort was completed by each respondent independently, either in isolation or in a group setting. Participants were also asked to complete a short survey including questions pertaining to demographics and previous experience with Web conferencing (Multimedia Appendix 3).

### Analysis

A by-person factor analysis of the Q-sort was conducted to identify groups of participants with similar viewpoints. Finally, for each factor a weighted (synthetic) Q-sort was produced using a weighted averaging method to calculate the score for each statement for that factor [24]. This synthetic Q-sort represents the set of responses to statements that are held by a person who typifies that particular standpoint. To generate the synthetic Q-sort, the statistical programs use only the scores for those participants who loaded on the factor. The scale of this synthetic Q-sort is basically a normalized Z-scale. However, these scores can easily be converted to the original Q-sort format, the two statements with the highest weighted composites being assigned +4, the next three highest being scored +3, and so forth. PQMethod (version 2.11) was used for the analysis of Q-sorts. PQMethod is a frequently used program developed by Schmolck [25] that can be downloaded freely from his website.

So far, only two methods of factor extraction are implemented in this program: principal component method and centroid method. In addition, only two methods of rotation are available in this program: varimax and judgmental (or manual) rotations. Usually, rotation methods are informed by theoretical reasoning rather than simply by statistical criteria. Interested readers are referred to the guidelines accompanying relevant software for practical guidance or to Brown [26] for a theoretical account. The main difference between principal component method and centroid method is that in principal component the variance of loadings is maximized, where in centroid the average of the loadings is maximized. Although no clear statistical or theoretical advantage is provided in Q-methodology literature, there is great sympathy among Q-methodologists for using the centroid method. We used the centroid method for factor extraction. All authors met as a group over a half day to interpret the factors; consensus was quickly reached in assigning a name to each factor and describing the viewpoint since the pattern of statements clearly pointed to unique and distinguishing views.

## Results

### Participants

A total of 36 people participated in the study. Each participant who completed the Q-sort had previous exposure to the technology, ranging from attendance at a Web conference (set up and managed by someone else such as a faculty member or technical support person) to being highly engaged (setting up, connecting, and actively participating in a Web conference from a remote location). Participants had connected as a group from their classroom or alone from a remote location such as an office or home (Table 1). One participant connected from South America.

**Table 1:** Description of participants (N = 36)

	**Number**	**Percentage**
**Participant**		
Medical residents in anesthesiology	14	39
Nursing graduate students	11	31
Health sciences faculty	9	25
Health sciences staff	2	6
**Gender**		
Male	12	33
Female	24	67
**Experience with Web conferencing**		
Participant	26	72
Guest presenter	2	6
Moderator	1	3
Supported others to run a Web conference	1	3
Multiple roles	6	17
Connectionpoints^*^		
Classroom	20	59
Office and other location	6	18
Home	5	15
Other	3	9

^*^There were two 2 responses missing for one question related to connection points.

Participants were asked about their experiences in setting up Web conferences, such as uploading a presentation or creating multiple-choice questions or polls. Of the 36 participants, 53% had never set up a Web conference (they arrived to a room where the setup was done for them); 22% had content set up by others; 14% had uploaded materials for a Web conference themselves; and 11% were unsure about their past experiences. Most participants were not developers of the technology but represented general users. Therefore, our sample included people with a range of experience in Web conferencing, which is typical of most situations when a new technology is introduced.

### Major Viewpoints

Three major viewpoints emerged from the Q-sort analysis, each of which presented generally positive opinions of Web conferencing; 34 participants loaded on three factors, which we labeled factor 1, pragmatists; factor 2A, positive communicators; and factor 2B, shy enthusiasts. These factors explained 28% (factor 1) and 11% (factor 2, including 2A and 2B) of the total variance, respectively. Although the total of 39% is less than what is seen in ordinary factor analysis, in Q-methodology the main objective is finding the preferences (or salient viewpoints), not identifying the number of factors that can explain a large percentage of the total variations. Two participants did not load on any of the factors.

The majority of our respondents (n = 26) loaded on factor 1, the pragmatists. Table 2 lists the scores for the distinguishing statements for this factor, which vary from −4 to +4, where negative scores indicate disagreement with the statement. In this group, there was strong agreement with four statements: (1) “Web conferencing provides students with flexibility to participate when off-site”; (2) “Although face-to-face meetings are better than Web conferencing, for those people who can’t be there, Web conferencing is useful”; (3) “There is potential for technical difficulties during Web-conferencing, which can jeopardize its effectiveness”; and (4) “There is potential for Web conferencing to support education.” They strongly disagreed with the statements “I am much less shy communicating from home, than I would be on-site!” and “I would prefer to attend seminars online rather than face-to-face for cost savings.” Therefore, this group of respondents endorsed the value of Web conferencing for its increased flexibility and easy access for distant participants while being realistic about problems with ease of use and potential technical difficulties that could jeopardize the experience. Although pragmatists generally preferred face-to-face meetings, they also felt that “if you can’t be there,” Web conferencing can be a useful technology.

**Table 2:** Factor 1 scores for distinguishing statements for pragmatists (n = 26)*

**Statement**	**PragmatistsFactor 1**	**Positive CommunicatorsFactor 2A**	**Shy EnthusiastsFactor 2B**
Web conferencing provides students with flexibility to participate when off-site.	**4**	2	−1
Although face-to-face meetings are better than Web conferencing, for those people who can’t be there, Web conferencing is useful.	**3**	0	−3
There is potential for technical difficulties during Web conferencing, which can jeopardize its effectiveness.	**3**	0	−1
There is potential for Web conferencing to support education.	**3**	0	0
I would prefer to attend seminars online rather than face-to-face for cost savings.	−**3**	0	4
I am much less shy communicating from home than I would be on-site!	−**4**	−1	3

^*^Negative scores denote disagreement with the statement.

Factor 2 was a bipolar factor, which implies that two opposite viewpoints, representing two groups of participants, loaded significantly on the same factor. A bipolar factor is typically broken down into two factors: one containing Q-sorts with positive loadings and the other containing Q-sorts with negative loadings. Therefore, we split factor 2 into factors each describing a common viewpoint (2A and 2B). Four respondents loaded on factor 2A (Table 3); they held a unique viewpoint represented by the theme of positive communicators. This group strongly agreed with three statements: (1) “Web conferencing can facilitate communication in research teams who are in multiple locations”; (2) “I enjoy trying out a new technology”; and (3) “Web conferencing would be useful to support the supervision of students in distributed locations.” They strongly disagreed with the statement “The application sharing tool is a bit confusing for participants and presenters.” Positive communicators generally enjoyed new technology and felt that Web conferencing could facilitate communication in a variety of contexts, including education and research team meetings. They were not challenged by the application-sharing feature in which a presenter opens a software application (eg, Internet Explorer, Excel) on their computer to provide a live demonstration to remote participants.

**Table 3:** Factor 2A scores for distinguishing statements for positive communicators (n = 4)*

**Statement**	**PragmatistsFactor 1**	**Positive CommunicatorsFactor 2A**	**Shy EnthusiastsFactor 2B**
Web conferencing can facilitate communication in research teams who are in multiple locations.	2	**4**	−2
I enjoy trying out a new technology.	0	**3**	1
Web conferencing would be useful to support the supervision of students in distributed locations.	1	**3**	2
The application sharing tool is a bit confusing for participants and presenters.	0	−**3**	1

^*^Negative scores denote disagreement with the statement.

The last four respondents loaded on factor 2B (Table 4); they held a viewpoint that could best be described as shy enthusiasts. This group of respondents strongly agreed with the following: (1) “I find Web conferencing software extremely easy to use”; (2) “I would prefer to attend seminars online rather than face-to-face for cost savings”; (3) “The ability to use multiple-choice questions and open-ended questions is a very important feature in Web conferencing”; and (4) “I am much less shy communicating from home than I would be on-site!” They disagreed with the statements “Although face-to-face meetings are better than Web conferencing, for those people who can’t be there, Web conferencing is useful,” and “Nonverbal communication in the classroom is missed by those online; this can cause confusion.” Shy enthusiasts clearly preferred Web conferencing to face-to-face seminars and were comfortable with the technology overall. The ability to interact online by responding to multiple-choice or open-ended questions was valued. In addition, they were less shy meeting from a remote location compared to face-to-face and did not feel disadvantaged with the lack of nonverbal communication cues when communicating online. During this study, no participants used the video feature. All four of these participants were anesthesiology residents.

**Table 4:** Factor 2B scores for distinguishing statements for shy enthusiasts (n = 4)*

**Statement**	**PragmatistsFactor 1**	**Positive CommunicatorsFactor 2A**	**Shy EnthusiastsFactor 2B**
I find Web conferencing software extremely easy to use.	−2	−1	**4**
I would prefer to attend seminars online rather than face-to-face for cost savings.	−3	0	**4**
The ability to use multiple-choice questions and open-ended questions is a very important feature in Web conferencing.	−2	0	**3**
I am much less shy communicating from home than I would be on-site!	−4	−1	**3**
Although face-to-face meetings are better than Web conferencing, for those people who can’t be there, Web conferencing is useful.	3	0	−**3**
Nonverbal communication in the classroom is missed by those online; this can cause confusion.	0	−2	−**4**

^*^Negative scores denote disagreement with the statement.

### Extreme Scores and Consensus Statements

Table 5 illustrates statements that yielded extreme scores that were not distinguishing statements but that can be of particular interest because they represent the most prominent likes and dislikes of the participants loaded on one factor [26]. For example, the statement “Web conferencing can enhance distance education through increased access to seminars, rounds, etc.” was given a high score by both pragmatists and positive communicators.

**Table 5:** Statements with extreme scores for each factor*

**Statement**	**PragmatistsFactor 1**	**Positive CommunicatorsFactor 2A**	**Shy EnthusiastsFactor 2B**
**Pragmatists, factor 1**			
Web conferencing can enhance distance education through increased access to seminars, rounds, etc.	**4**	4	0
I feel very involved when I am in a Web conference.	−**3**	2	2
**Positivecommunicators, factor 2A**			
Web conferencing can enhance distance education through increased access to seminars, rounds, etc.	4	**4**	0
The audio feature is a very important function of Web conferencing.	2	**3**	−1
I experience extensive anxiety about my ability to set up the teleconference and more anxiety about my ability to “troubleshoot” in the middle of a session.	−2	−**3**	1
Lack of video is an issue.	−1	−**3**	−2
**Shy enthusiasts, factor 2B**			
Overall, the quality of Web conferencing technology is very good.	−1	2	**3**

^*^Negative scores denote disagreement with the statement.

Pragmatists did not feel as highly involved when taking part in a Web conference compared to positive communicators and shy enthusiasts. Like positive communicators, however, they felt strongly that Web conferencing could enhance distance education by providing increased access to various educational offerings such as seminars and rounds. Positive communicators expressed less anxiety about setting up Web technology, valued the audio feature more, but missed the video component less than the other groups. Finally, shy enthusiasts felt more strongly that the overall quality of Web conferencing was very good. All groups generally agreed that the video feature was unimportant; however, it should be noted that the video feature was not used during the trial due to its poor quality.

Although all three groups had different viewpoints on a number of aspects of Web conferencing, there was consensus on several statements (Table 6). All participants felt strongly that students would enjoy Web conferencing and agreed that more training would be useful. They also disagreed with the idea that Web conferencing would hamper their interactivity online and that the technology ran slowly. Participants from all three groups felt that Web conferencing was superior to audio conferencing alone.

**Table 6:** Consensus statements*

**Statement**	**PragmatistsFactor 1**	**Positive CommunicatorsFactor 2A**	**Shy EnthusiastsFactor 2B**
The PowerPoint slide presentation function is very important for Web conferencing.	2	1	1
Web conferencing is a more interesting way to connect people at a distance than audio conferencing.	1	2	1
More Web conferencing training sessions are necessary.	1	1	2
Web conferencing technology is not always compatible with the computer resources I have at home.	0	−1	0
The ability to record and archive seminars is extremely convenient for people who are unable to attend scheduled presentations.	1	1	0
I think Web conferencing would be useful for workshops/training for staff at their workstations.	−1	0	0
Web conferencing runs slowly.	−2	−2	−3
I did not feel that Web conferencing promoted interactivity with those people located at remote sites.	−3	−4	−4
Students accustomed to face-to-face learning will not enjoy Web conferencing experiences.	−4	−4	−3

^*^Negative scores denote disagreement with the statement.

Of females who loaded on factors, 86% (19/22) were pragmatists compared to 58% (7/12) of all males. The two administrative staff who participated in the study worked with Web conferencing the most. They were involved in setup and providing service/support. Both fell into the pragmatist group. Pragmatists also tended to include participants with varied Web conferencing experience, such as being a guest presenter and/or a moderator, providing support to others, as well as being a general participant.

## Discussion

### Web Conferencing in Education

With the increasing application of Web conferencing technologies in education, the results from our study provide an important contribution to understanding general users’ viewpoints on the role of Web conferencing as a synchronous communication system to support health sciences education. Based on our participants’ positive viewpoints on Web conferencing in health sciences education, a decision was made to continue to fund Web conferencing in the faculty. Their positive views were similar to other reports in the literature [11-13]. The most cautious/circumspect group was the pragmatists, who made up 72% of the total participants. They tended to have more varied hands-on experience with Web conferencing and thus were more likely to have experienced problems that can occur, thereby influencing their “pragmatic” views. As was pointed out in papers by Ostrow and DiMaria-Ghalili [13] and Shield et al [11], there are technical issues with Web conferencing that educators and technicians need to overcome, such as bandwidth limitations, firewall and security filters that block access, audio quality and screen sizing issues, and problems installing a client needed to run the Web conferencing software. We also experienced these issues at our site. Despite the problems, positive communicators envisioned the use of this technology for broader applications, including research and administration.

Our participants felt that more training would be useful. As others have identified, it is important to provide greater faculty orientation to ensure that minimal technical support is needed [12]. This has implications for the implementation of Web conferencing within faculty of health sciences programs, which often have many instructors who make only occasional teaching contributions. The steep learning curve for moderators and participants necessitates more technical support for occasional moderators. This is a useful caveat with regard to rolling out various features of Web conferencing since we observed that moderators generally used few features in their initial Web conferences but were more likely to introduce additional features, such as polling, in subsequent conferences.

It is somewhat surprising that learners were generally positive about Web conferencing given the lack of faculty training in instructional design methodologies and best practices for synchronous e-learning. While some moderators used the occasional interactive component such as polling, most moderators provided simple audio commentary of bulleted text slides. It is possible that improved use of Web conferencing best practices—such as using meaningful visuals, multimedia, and interactions like polling, application sharing, and chats—may have resulted in even more enthusiasm for the technology [29]. As with most pedagogical interventions, the quality of the instructional methods is more important than the medium or the technology itself [30].

The shy enthusiasts group may have comprised participants who experience social anxiety. It should be noted that although shy enthusiasts found the technology very easy to use, they were all attendees at a Web conference and did not have to set up or configure computers themselves. They participated as a group in a classroom with a faculty member presenting remotely. Although the four participants in the shy enthusiast group happened to be anesthesia residents, anesthesia residents also fell into the other two groups. Shyness has not been found to be related to use of chat rooms or email [31], but it has been found to be related to higher Internet use [32].

Our findings show that while Web conferencing was preferred over teleconferencing alone, the video feature was not highly valued by participants. This finding is supported in a study of two groups of dental students in Michigan who used podcasting to listen to lectures via audio alone or audio synchronized with PowerPoint and video [33]. The students reported a high preference for audio alone; they used the archives of lectures to review material after class and before exams and also as a safety net when they could not keep up with note-taking in information-dense classes. Although podcasting is an asynchronous communication system, it is not clear if the results are transferable to a synchronous context. Others have indicated that they have experienced problems with video caused by low bandwidth [12]. As the quality and ease of use of video delivery over the Web to personal computers improves, it is expected that its use will likely increase. Further studies to examine best practices for the application of video to support synchronous communication are needed. In the meantime, there appears to be good acceptance of audio and text alone.

### Q-Methodology in Educational Research

Q-methodology proved to be a useful and unique approach to investigating this educational research topic as the study benefited from both qualitative and quantitative perspectives. The validity of our interpretation of the results relies on the use of factor analysis in extracting the distinguishing statements and on the use of domain experts in interpretation. The accuracy of the interpretation could be further verified by asking the relevant (significantly factor-loaded) participants to comment on their views about the results of the study, although we could not conduct follow-up interviews. Despite this limitation, we had adequate numbers in our sample for the use of Q-methodology, as seen by the emergence of three clear and distinct factors. Perhaps our findings would have differed if the contexts for the use of Web conferencing were more varied. There were other applications of Web conferencing used during our trial, such as for support of multi-site research and pan-Canadian and international meetings. This evaluation did not focus on such applications, although some statements were included that referred to broader technology applications. Our study focused on the opinions of participants who were general users of the technology to support educational needs of health sciences students as opposed to experts. As ease of use and quality of Web conferencing increase, we might expect to find even more positive responses from participants in academic settings.

### Conclusion

This study contributes new knowledge about general users’ (faculty, student, and staff) viewpoints on Web conferencing technology as a support for health sciences education (see Multimedia Appendix 4 for a PowerPoint presentation of the study). All participants felt positively about the use of Web conferencing to support education, but for different reasons. Furthermore, there were no strongly negative views, thereby providing support for continued growth of such technologies in academia. Participants viewed Web conferencing as an enabler, especially where face-to-face meetings were not possible. Audio features of the software were highly valued, while video features were not particularly missed. Our findings indicate that adequate technical support and training must be provided for successful ongoing implementation of Web conferencing. More research is needed to determine best practices for the use of Web conferencing in various educational contexts. Our promising results provide an impetus for the continued application of Web conferencing technology to facilitate the delivery of health sciences education programs. Q-methodology was a useful research approach and is suggested in future exploration of the use of Web technologies to facilitate communication in education, research, and administration activities.

## Multimedia Appendix 1

Final statements for the Q-sort
							

## Multimedia Appendix 2

Instructions for participants completing the Q-sort
							

## Multimedia Appendix 3

Demographic survey
								

## Multimedia Appendix 4

PowerPoint presentation of study results
								
