# Supplementary material for: Pragmatists, Positive Communicators, and Shy Enthusiasts: Three Viewpoints on Web Conferencing in Health Sciences Education
Source: J Med Internet Res. 2007 Dec 31;9(5):e39. doi: 10.2196/jmir.9.5.e39 (PMC2270418; doi:10.2196/jmir.9.5.e39)
Supplement: Supplementary file 1 [file jmir_v9i5e39_app1.pdf]

Final Statements for Q-Sort

There is potential for web conferencing to support education.
I enjoy trying out a new technology.
Web conferencing provides students with flexibility to participate when off-site.
Overall, the quality of web conferencing technology is very good.
Web-conferencing increases my efficient use of time.
I love being able to hear a live presentation from the comfort of my home.
Web conferencing can enhance distance education through increased access to seminars, rounds, etc.
Web conferencing would be useful to support the supervision of students in distributed locations.
Web-conferencing can facilitate communication in research teams who are in multiple locations.
I think web conferencing would be useful for workshops/training for staff at their workstations.
I feel very involved when I am in a web conference.
I am much less shy communicating from home, than I would be on-site!
Although face-to-face meetings are better than web conferencing, for those people who can't be there, web conferencing is useful.
I would prefer to attend seminars online rather than face-to-face for cost savings.
The ability to record and archive seminars is extremely convenient for people and who are unable to attend scheduled presentations.
The interactive polling feature of web conferencing is great!
I find web conferencing software extremely easy to use.
Web conferencing is a more interesting way to connect people at a distance than audio conferencing.
The ability to use multiple-choice questions and open-ended questions is a very important feature in web conferencing.
The PowerPoint™ slide presentation function is very important for web conferencing.
The application sharing feature is a very important function of web conferencing.
Text chat is a very important function of web conferencing.
The audio feature is a very important function of web conferencing.
Web conferencing is likely to get easier to use with time.
Presenters must set up their materials early for a web conferencing session to run smoothly.
All presenters and participants must have the appropriate access to the equipment they will be using (i.e. microphone, speakers/headset, etc.).
More web conferencing training sessions are necessary.
Web conferencing runs slowly.
There is potential for technical difficulties during web-conferencing, which can jeopardize its effectiveness.
Web conferencing technology is not always compatible with the computer resources I have at home.
Setting up and maintaining web conferences was not as user friendly or intuitive as had been anticipated.
I experience extensive anxiety about my ability to set up the teleconference and more anxiety

about my ability to 'trouble shoot' in the middle of a session.
Lack of video is an issue.
Web conferencing could greatly benefit from a stronger video component.
Non-verbal communication in the classroom is missed by those on-line; this can cause confusion.
Students accustomed to face-to-face learning will not enjoy web-conferencing experiences.
I did not feel that web conferencing promoted interactivity with those people located at remote sites.
There is reduced comfort in using the technology remotely, if you are not a competent typist.
Web conferencing at the beginning is extremely resource intensive, especially if few individuals are connecting from off-site.
There are problems with audio quality.
It is somewhat challenging to deliver the presentation and address questions from live audience, while keeping in-tune with virtual audience and their questions or statements.
The application sharing tool is a bit confusing for participants and presenters.
