# Supplementary material for: Pragmatists, Positive Communicators, and Shy Enthusiasts: Three Viewpoints on Web Conferencing in Health Sciences Education
Source: J Med Internet Res. 2007 Dec 31;9(5):e39. doi: 10.2196/jmir.9.5.e39 (PMC2270418; doi:10.2196/jmir.9.5.e39)
Supplement: Supplementary file 2 [file jmir_v9i5e39_app2.pdf]

Web Conferencing Evaluation Instructions
FHS McMaster University
Research Project: Use of Q-Methodology to Examine Faculty and Student Experience With Web Conferencing Technology in Health Sciences Education

Thank you for participating in our project, which is designed to identify and explore faculty and student experience in using web conferencing in health sciences education.

Instructions:

Please read all of the instructions before beginning the Q-Sort Exercise.

PART A (Questionnaire)

Please complete the demographic questionnaire.

PART B (The Q-Sort)

Please note that this section is a 3-Step Process.

Step 1: This section is a sorting process of the 42 statement cards that have been distributed to you.

You have been given a list of 42 statement cards. Please divide the 42 statement cards into three categories: agree, disagree, and neutral. *Note: you are free to change your arrangement of statements at any stage in this process.*
By the end of this stage you should have 17 statement cards in your agree category, 17 statement cards in your disagree category, and 8 in your neutral category.

Example:

**Categories**	*Disagree*	*Neutral*	*Agree*
**Number of Statement Cards**	*17 Cards*	*8 Cards*	*17 Cards*

Step 2:

In the next section, you will be asked to distribute your statement cards from -4 (Strongly Disagree) through 0 (Neutral) to +4 (Strongly Agree). ***REMEMBER: you are free to change your arrangement of statements at any stage in this process.***

Example:

**-4**	**-3**	**-2**	**-1**	**0**	**+1**	**+2**	**+3**	**+4**
**2 Cards**	**3 Cards**	**5 Cards**	**7 Cards**	**8 Cards**	**7 Cards**	**5 Cards**	**3 Cards**	**2 Cards**

Sorting from each of the categories

Choose the **two statements** from the **'Agree Category'** that you most strongly agree with and place these under the +4 marker card.
Choose the **three statements** from the remaining cards that you most strongly agree with and place these under the +3 marker card.
Choose the **five statements** from the remaining cards that you most strongly agree with and place these under the +2 marker card.
Choose the **seven statements** from the remaining cards that you most strongly agree with and place these under the +1 marker card.
Choose the **two statements** from the **'Disagree Category'** that you most strongly disagree with and place these under the -4 marker card.
Choose the **three statements** from the remaining cards that you most strongly disagree with and place these under the -3 marker card.
Choose the **five statements** from the remaining cards that you most strongly disagree with and place these under the -2 marker card.
Choose the **seven statements** from the remaining cards that you most strongly disagree with and place these under the -1 marker card.
Place the **remaining eight items** that you feel most **neutral** about under the 0 'Marker Card'.

Step 3: Recording Your Answers

On the back of each statement card is a number.
Use the *Q-Sort Table* (on the reverse side of the *Demographic Questionnaire*) to record the number assigned to each statement item.

Example: Q-Sort Table

Strongly Disagree Strongly Agree

-4	-3	-2	-1	0	+1	+2	+3	+4

EXAMPLE

Thank you for participating in our project, which will result in valuable information about the use of Web conferencing in education.
