# Supplementary material for: Pragmatists, Positive Communicators, and Shy Enthusiasts: Three Viewpoints on Web Conferencing in Health Sciences Education
Source: J Med Internet Res. 2007 Dec 31;9(5):e39. doi: 10.2196/jmir.9.5.e39 (PMC2270418; doi:10.2196/jmir.9.5.e39)
Supplement: Supplementary file 3 [file jmir_v9i5e39_app3.pdf]

McMaster University
Faculty of Health Sciences Web Conference Evaluation
Demographic Survey

1. Which best describes you? (Check one)
   1. Undergraduate student
   2. Graduate student
   3. Medical resident
   4. Faculty member
   5. Staff member

2. What is your discipline? (Check one)
   1. Medicine
   2. Midwifery
   3. Nursing
   4. Occupational Therapy
   5. Physiotherapy
   6. Other, Please explain

3. What is your gender?
   1. Female
   2. Male

4. Which describes your past role/s in web conferencing (any web conferencing software e.g. WebEx, Horizon Wimba Live Classroom)? (Check all that apply)

I have been a:
   1. Participant in a web conference
   2. Guest presenter in a web conference.
   3. Moderator of a web conference.
   4. Supporting others to run a web conference

5 . Which best describes the context of your involvement with web conferences in the past? (Check all that apply)
I have been involved in a web conference/s to:

   1. Support education.
   2. Support research.
   3. Support administrative functions.
   4. Support other function/s.
      Explain:__________________________________

6. Which best describes your experiences in connecting to a web conference. (Check all that apply)

1. I have connected to a web conference from my office.
2. I have connected to a web conference from a classroom.
3. I have connected to a web conference from my home.
4. I have connected to a web conference from another location (Please explain). ____

7. Have you ever configured your computer (running the set up wizard on your computer) to participate in a web conference using Horizon Wimba's Live Classroom?

1. Yes
2. No
3. Not sure

8. Which best describes your experiences in setting up a web conference (uploading content, creating multiple choice questions, etc.).

1. I have never set up a web conference.
2. The content for my web conference was set up for me by others, once I sent in my materials.
3. I have uploaded web conference materials (uploaded PowerPoint™ or created multiple choice questions) in preparation for a web conference.
4. I am not sure.

9. Approximately how many web conferences have you participated in (using any web conferencing software e.g. Horizon Wimba Live Classroom, WebEx)?

____

FACULTY ONLY

10. How many years have you been teaching? _________ Years

11. What is your status as a faculty member? (Check all that apply).

1. Full-Time
2. Part-Time
3. Level Chair / Coordinator
4. Course Planner
5. Clinical Appointment
6. Senior Administrator (Dean, Assistant Dean)

Thank you! See back of page for Q-Sort Table

Strongly Disagree **Strongly Agree**

-4	-3	-2	-1	0	+1	+2	+3	+4
