# Supplementary material for: Pragmatists, Positive Communicators, and Shy Enthusiasts: Three Viewpoints on Web Conferencing in Health Sciences Education
Source: J Med Internet Res. 2007 Dec 31;9(5):e39. doi: 10.2196/jmir.9.5.e39 (PMC2270418; doi:10.2196/jmir.9.5.e39)
Supplement: Supplementary file 4 [file jmir_v9i5e39_app4.ppt]

## Slide 1
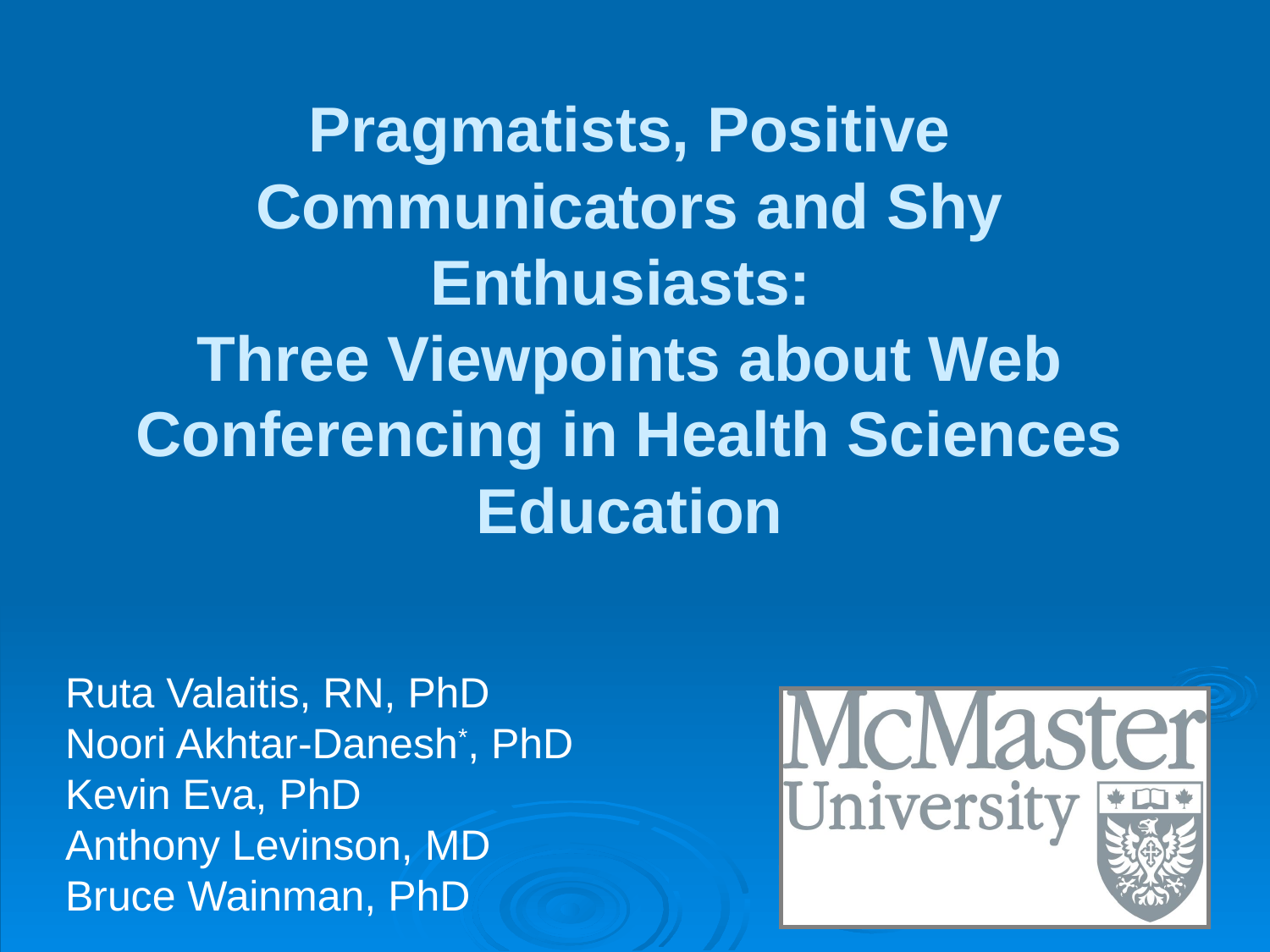

# Pragmatists, Positive Communicators and Shy Enthusiasts: Three Viewpoints about Web Conferencing in Health Sciences Education
Ruta Valaitis, RN, PhD
Noori Akhtar-Danesh*, PhD
Kevin Eva, PhD
Anthony Levinson, MD
Bruce Wainman, PhD

## Slide 2
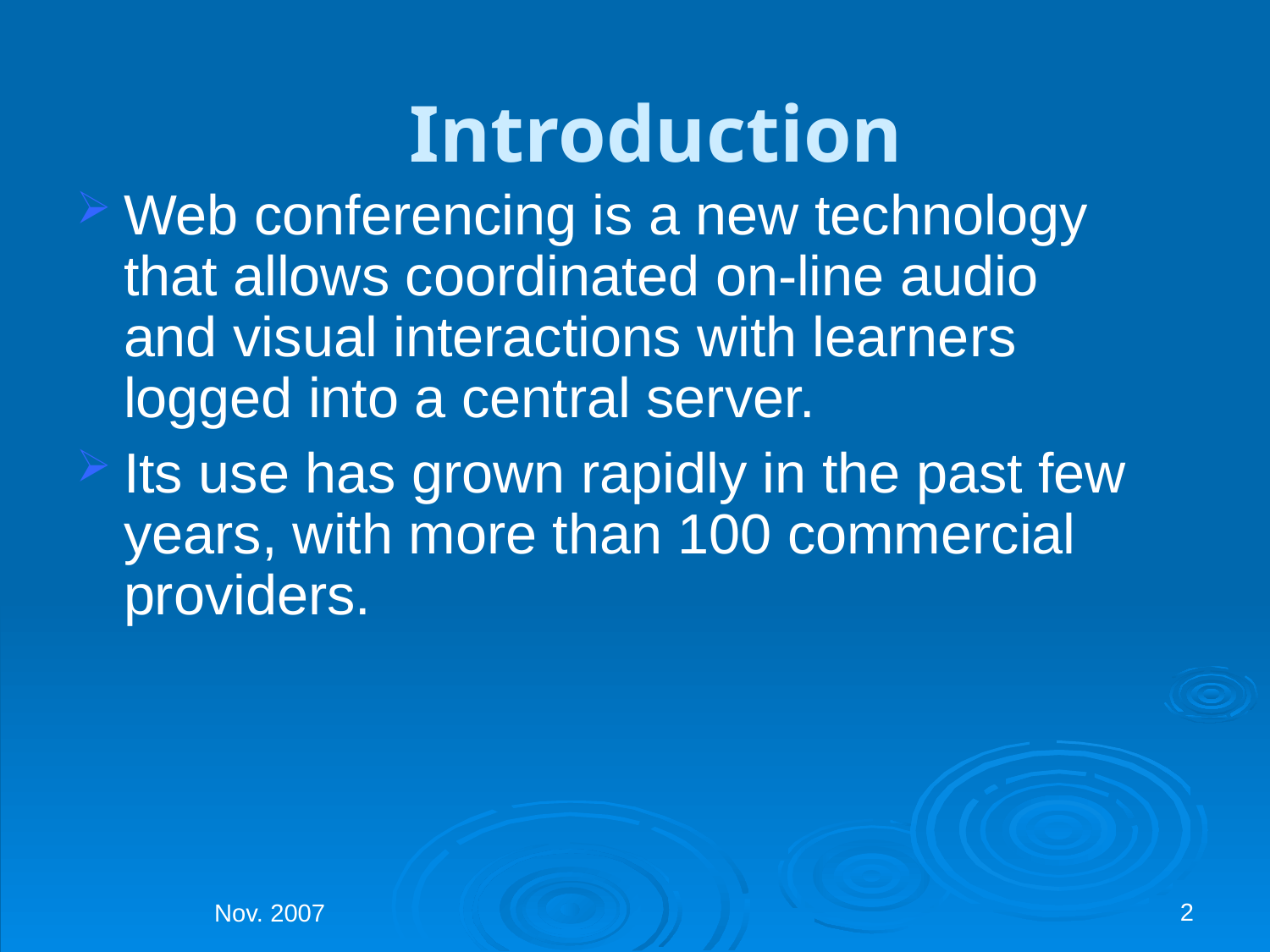

# Introduction
Web conferencing is a new technology that allows coordinated on-line audio and visual interactions with learners logged into a central server.
Its use has grown rapidly in the past few years, with more than 100 commercial providers.
2
Nov. 2007

## Slide 3
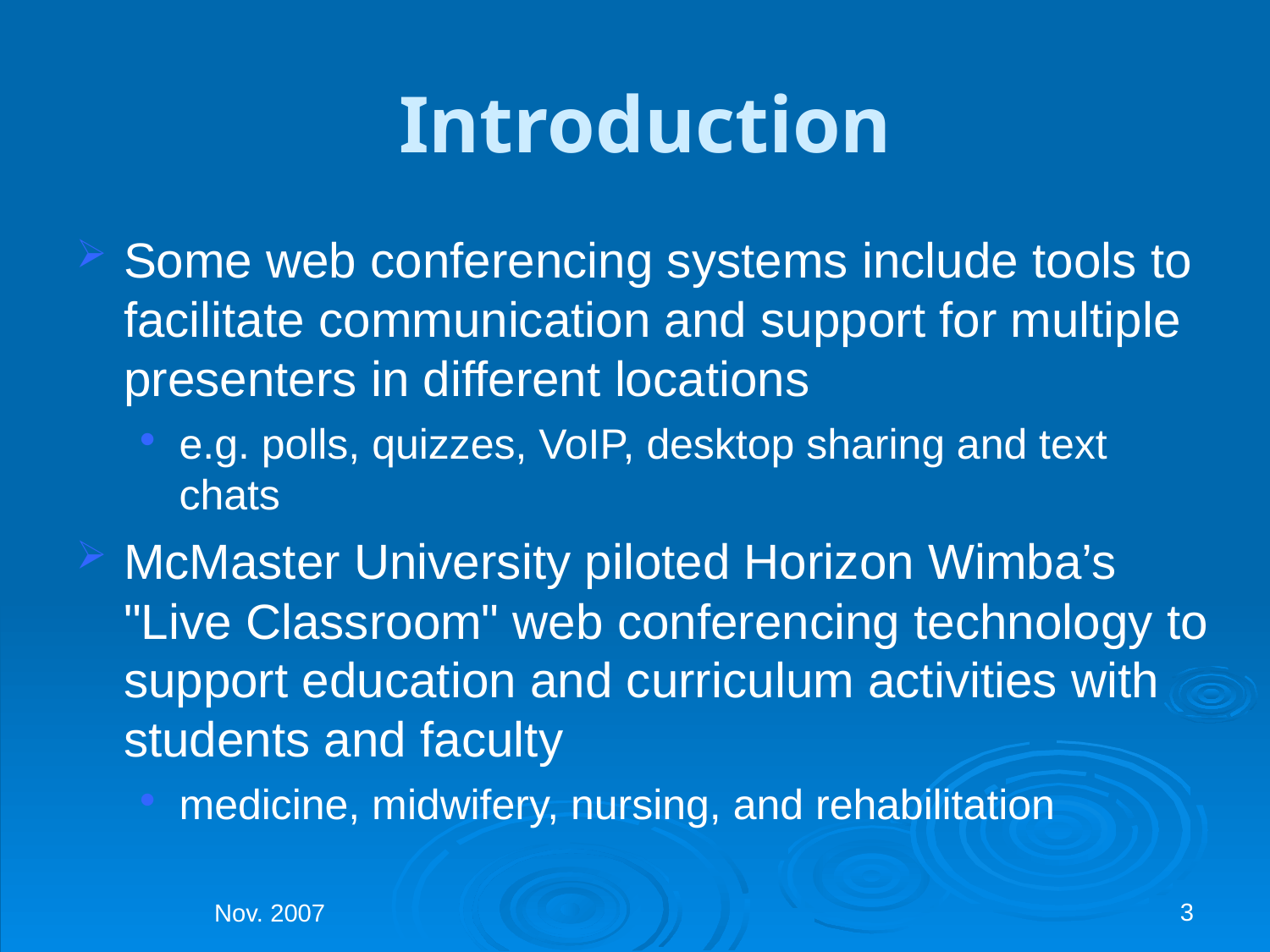

# Introduction
Some web conferencing systems include tools to facilitate communication and support for multiple presenters in different locations
e.g. polls, quizzes, VoIP, desktop sharing and text chats
McMaster University piloted Horizon Wimba’s "Live Classroom" web conferencing technology to support education and curriculum activities with students and faculty
medicine, midwifery, nursing, and rehabilitation
3
Nov. 2007

## Slide 4
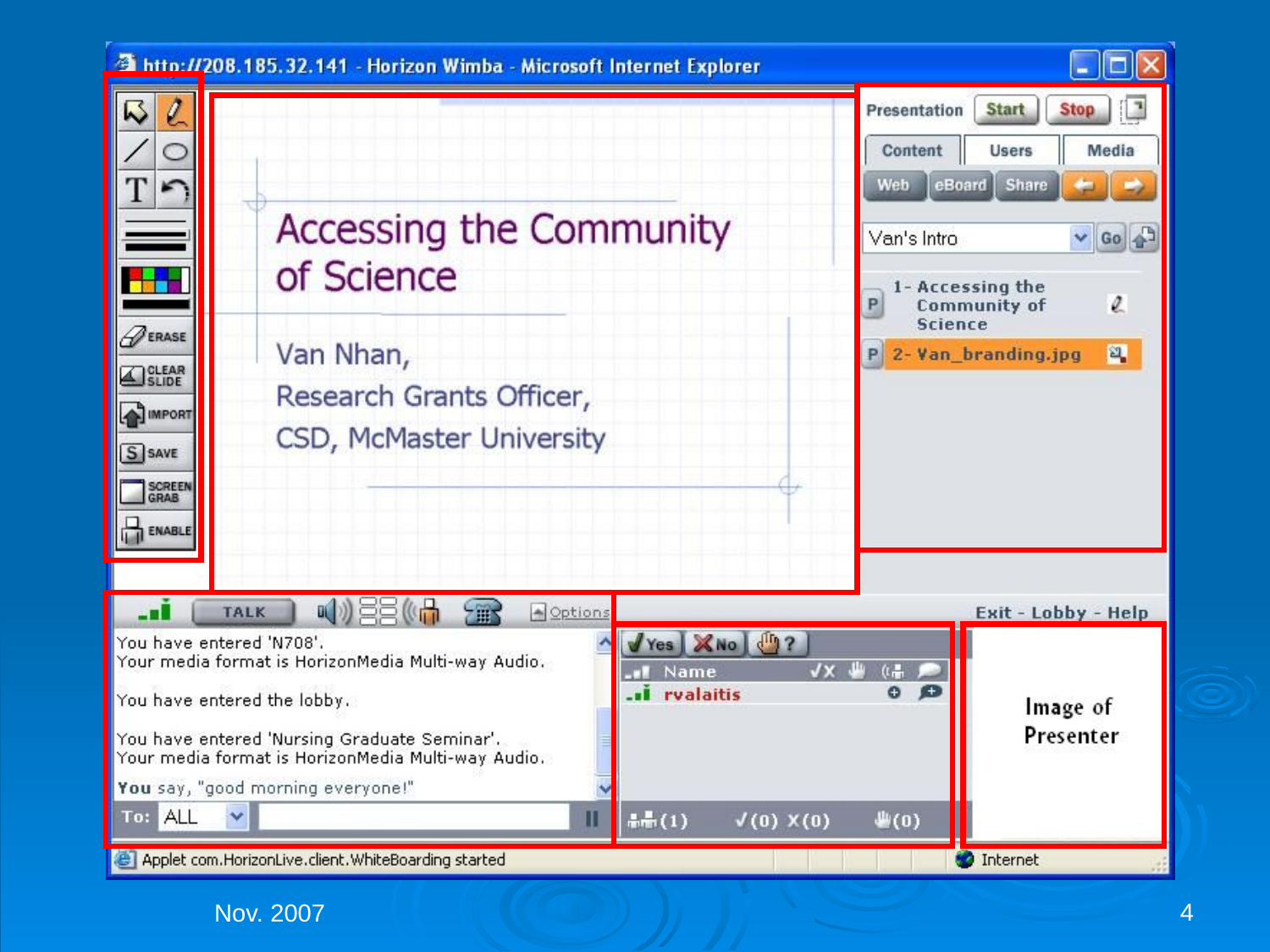

4
Nov. 2007

## Slide 5
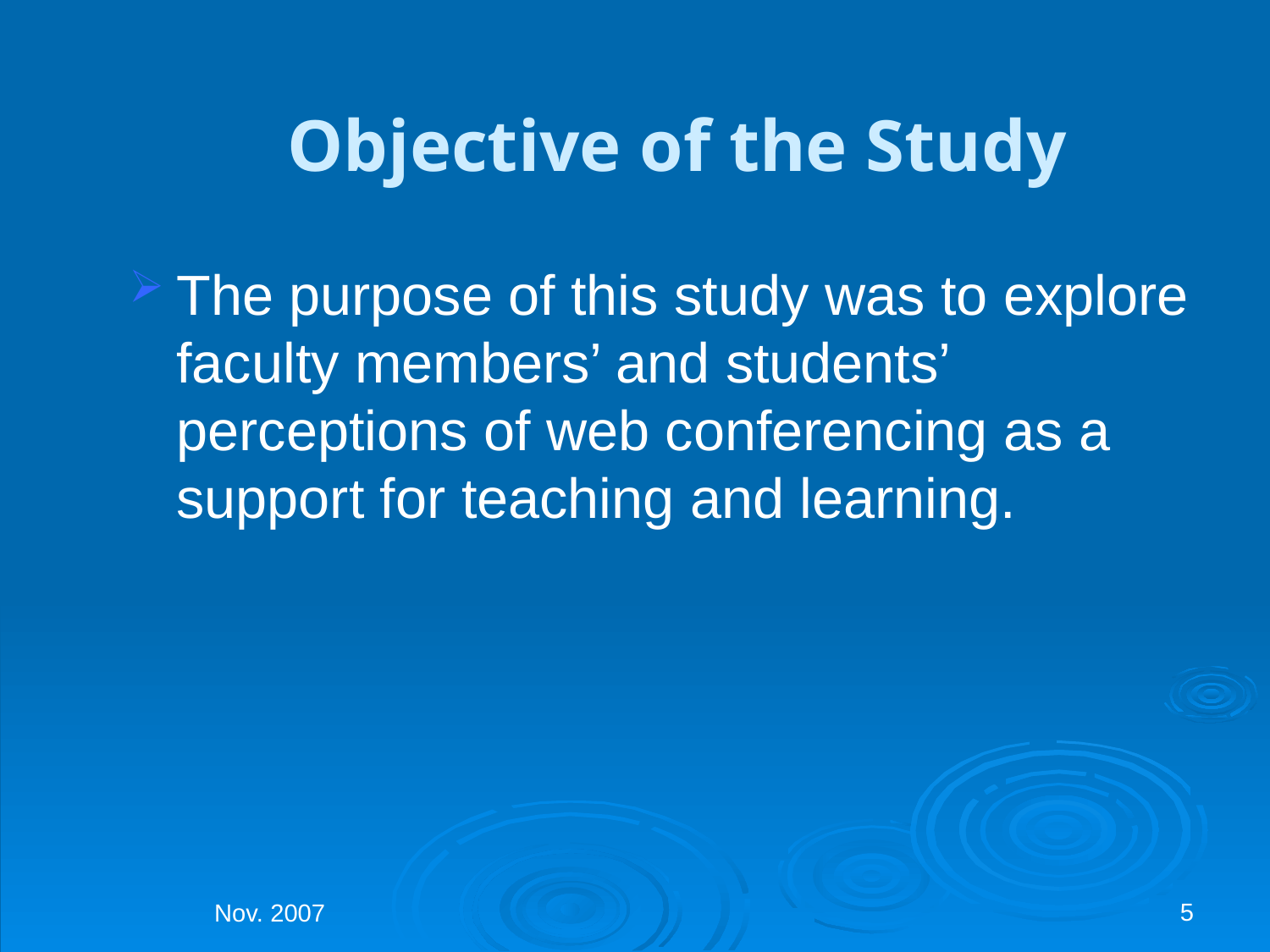

# Objective of the Study
The purpose of this study was to explore faculty members’ and students’ perceptions of web conferencing as a support for teaching and learning.
5
Nov. 2007

## Slide 6
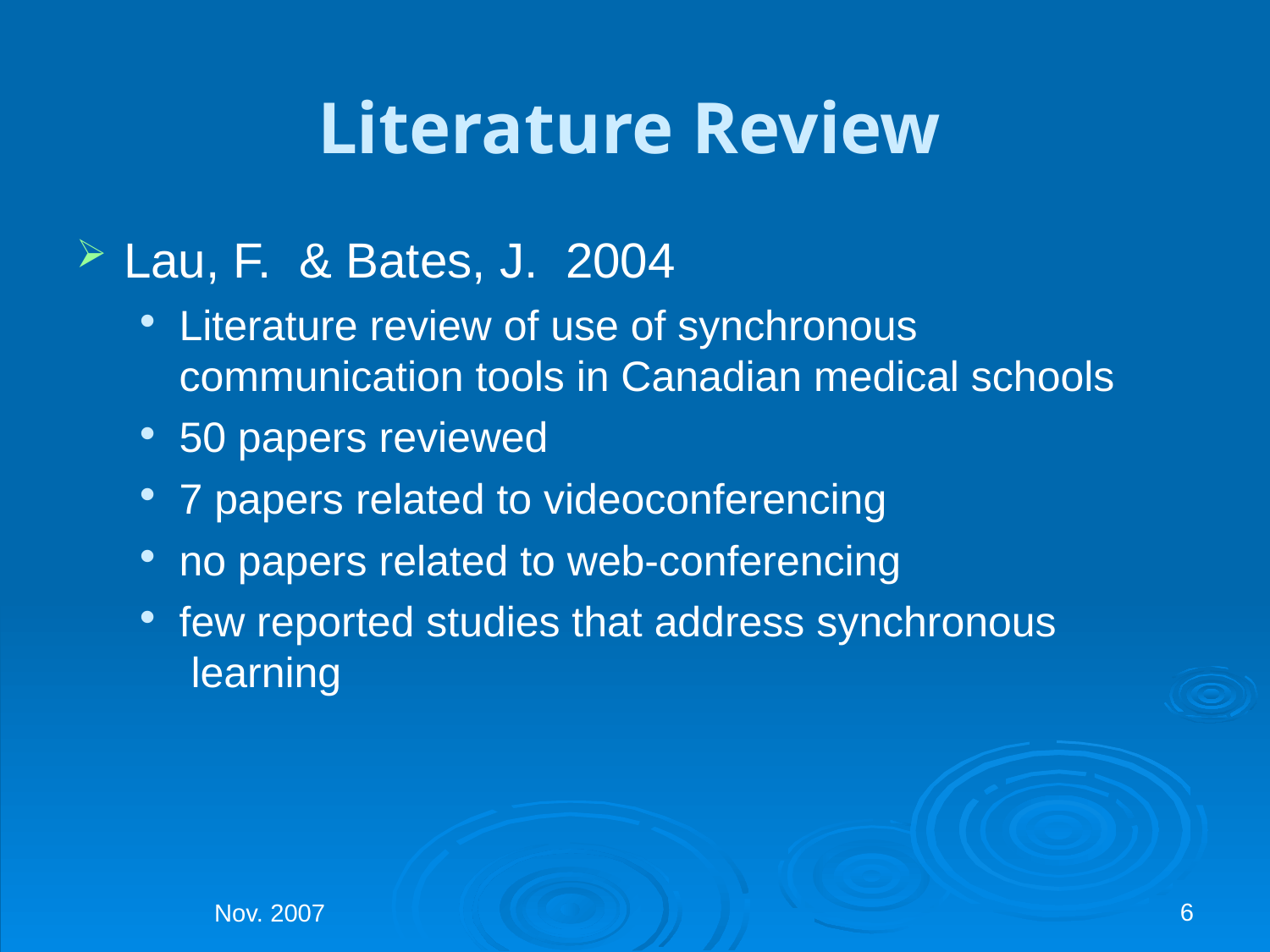

# Literature Review
Lau, F. & Bates, J. 2004
Literature review of use of synchronous communication tools in Canadian medical schools
50 papers reviewed
7 papers related to videoconferencing
no papers related to web-conferencing
few reported studies that address synchronous  learning
6
Nov. 2007

## Slide 7
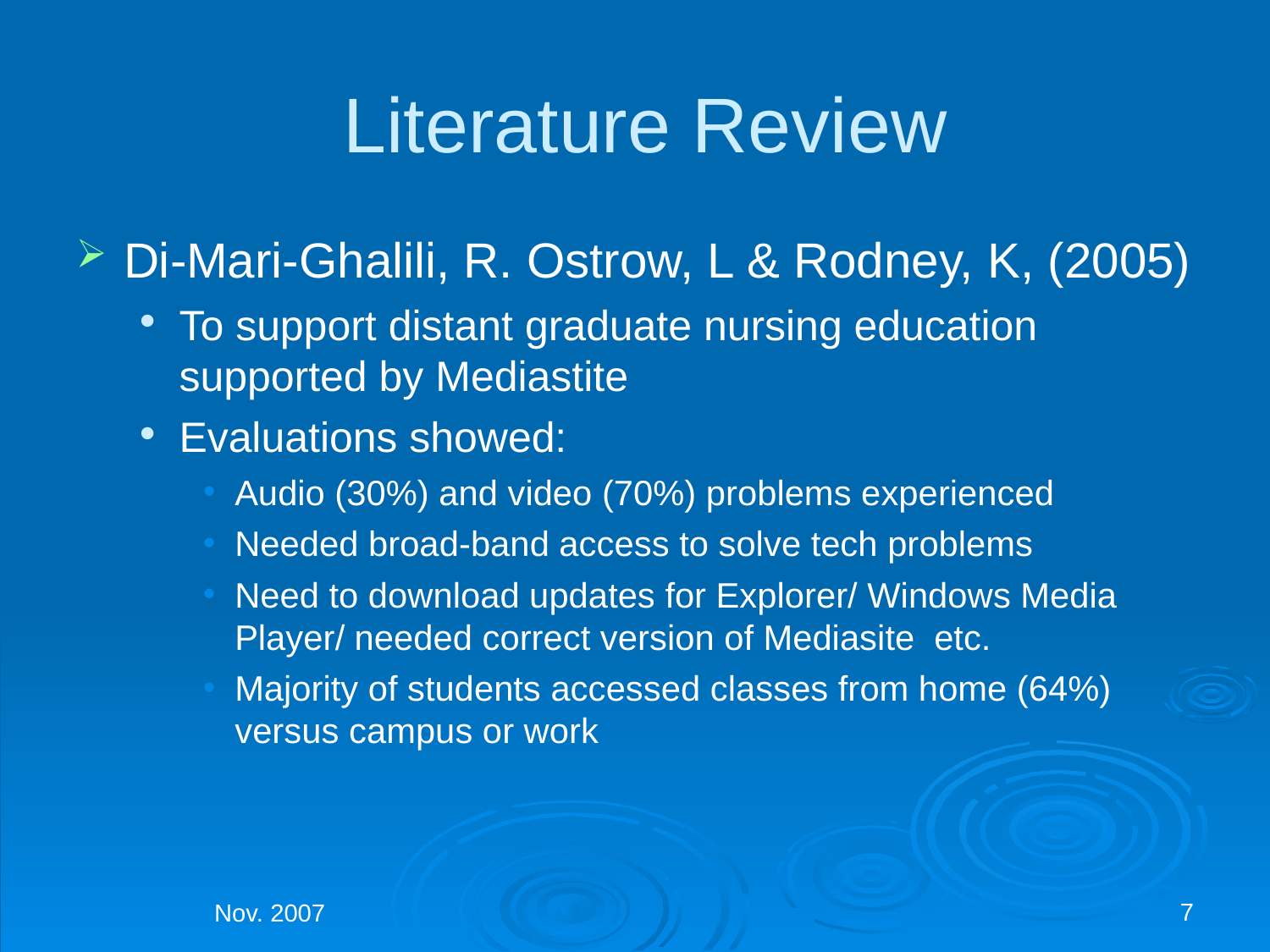

# Literature Review
Di-Mari-Ghalili, R. Ostrow, L & Rodney, K, (2005)
To support distant graduate nursing education supported by Mediastite
Evaluations showed:
Audio (30%) and video (70%) problems experienced
Needed broad-band access to solve tech problems
Need to download updates for Explorer/ Windows Media Player/ needed correct version of Mediasite  etc.
Majority of students accessed classes from home (64%) versus campus or work
7
Nov. 2007

## Slide 8
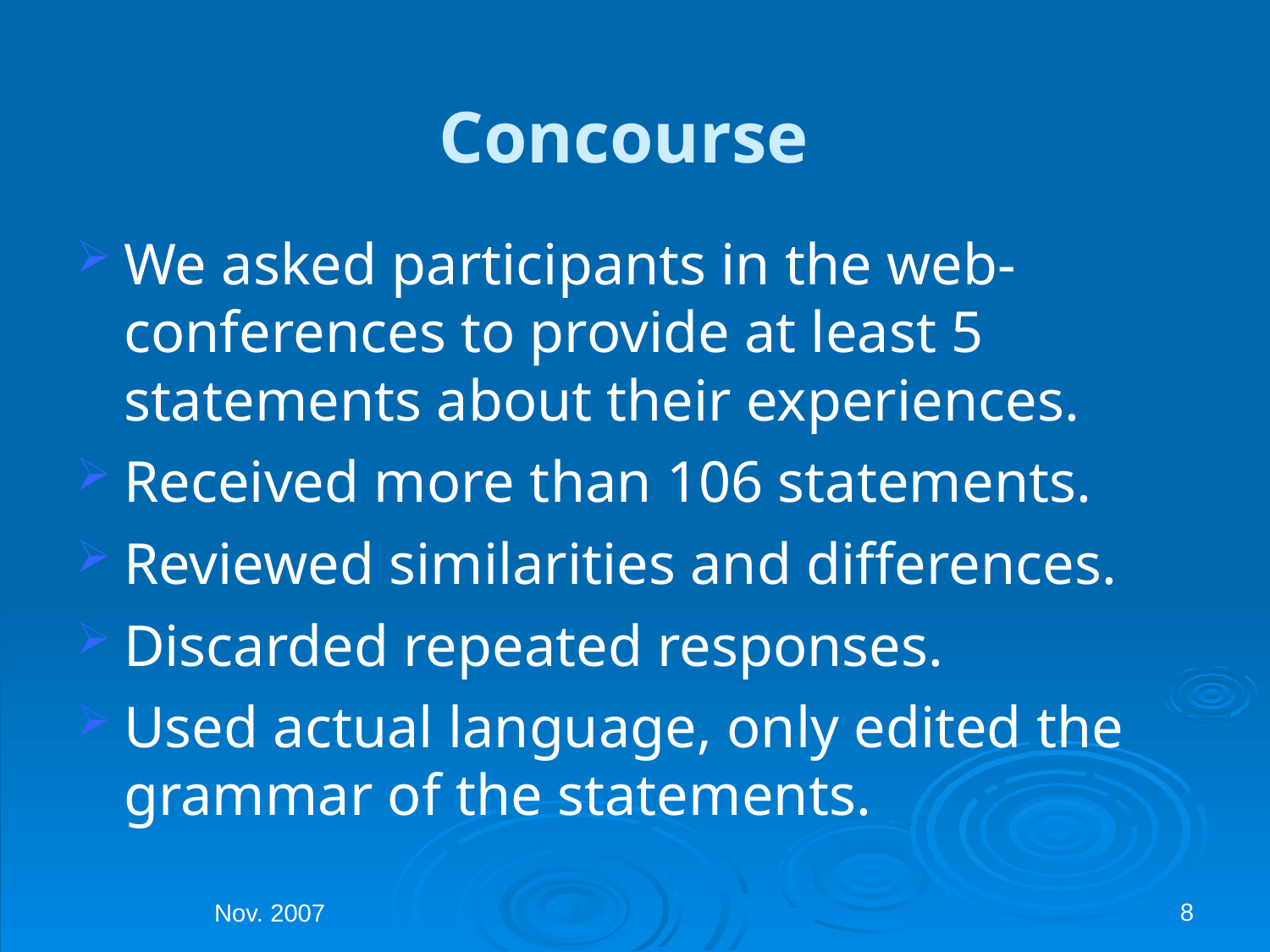

# Concourse
We asked participants in the web-conferences to provide at least 5 statements about their experiences.
Received more than 106 statements.
Reviewed similarities and differences.
Discarded repeated responses.
Used actual language, only edited the grammar of the statements.
8
Nov. 2007

## Slide 9
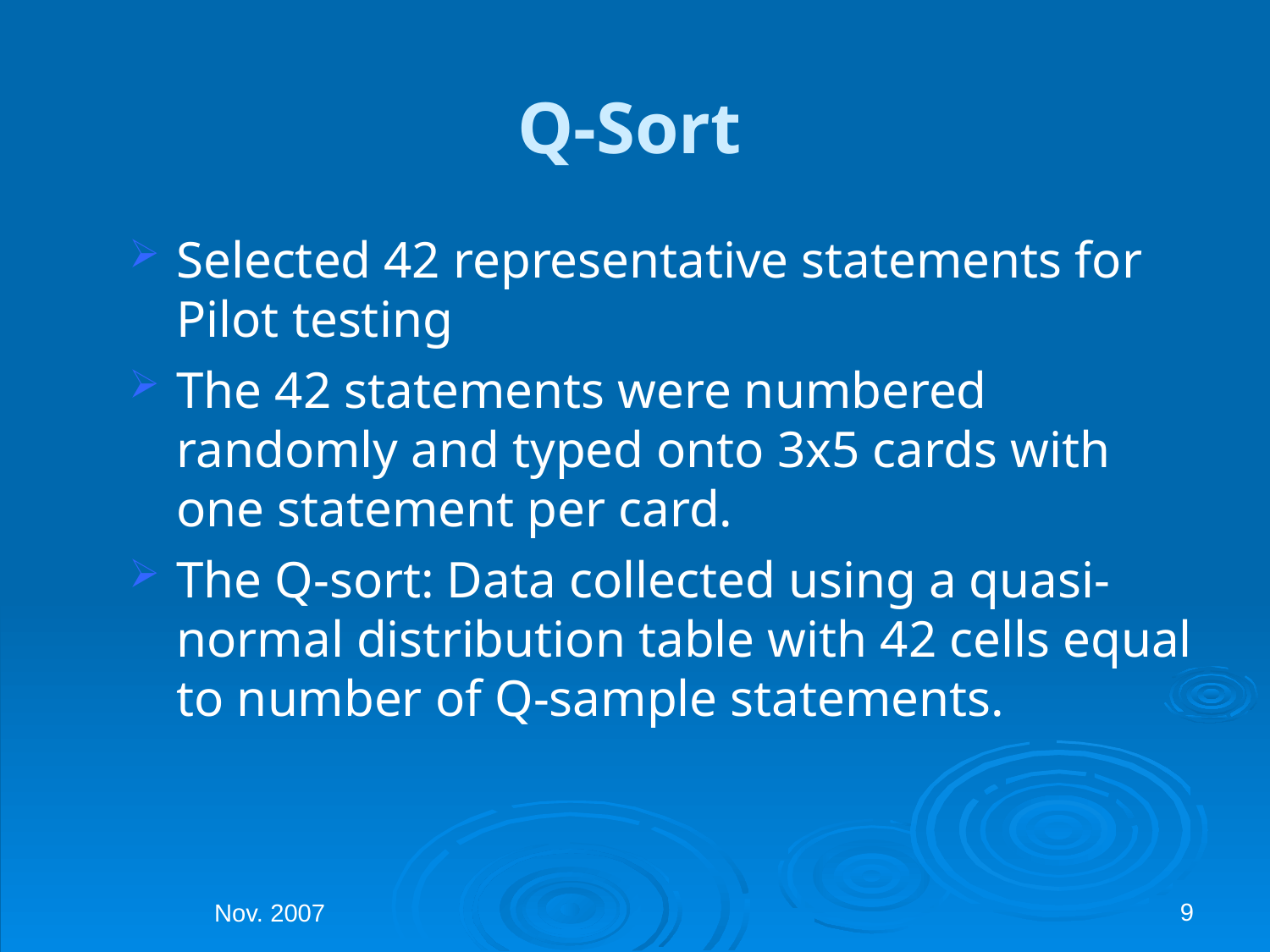

# Q-Sort
Selected 42 representative statements for Pilot testing
The 42 statements were numbered randomly and typed onto 3x5 cards with one statement per card.
The Q-sort: Data collected using a quasi-normal distribution table with 42 cells equal to number of Q-sample statements.
9
Nov. 2007

## Slide 10
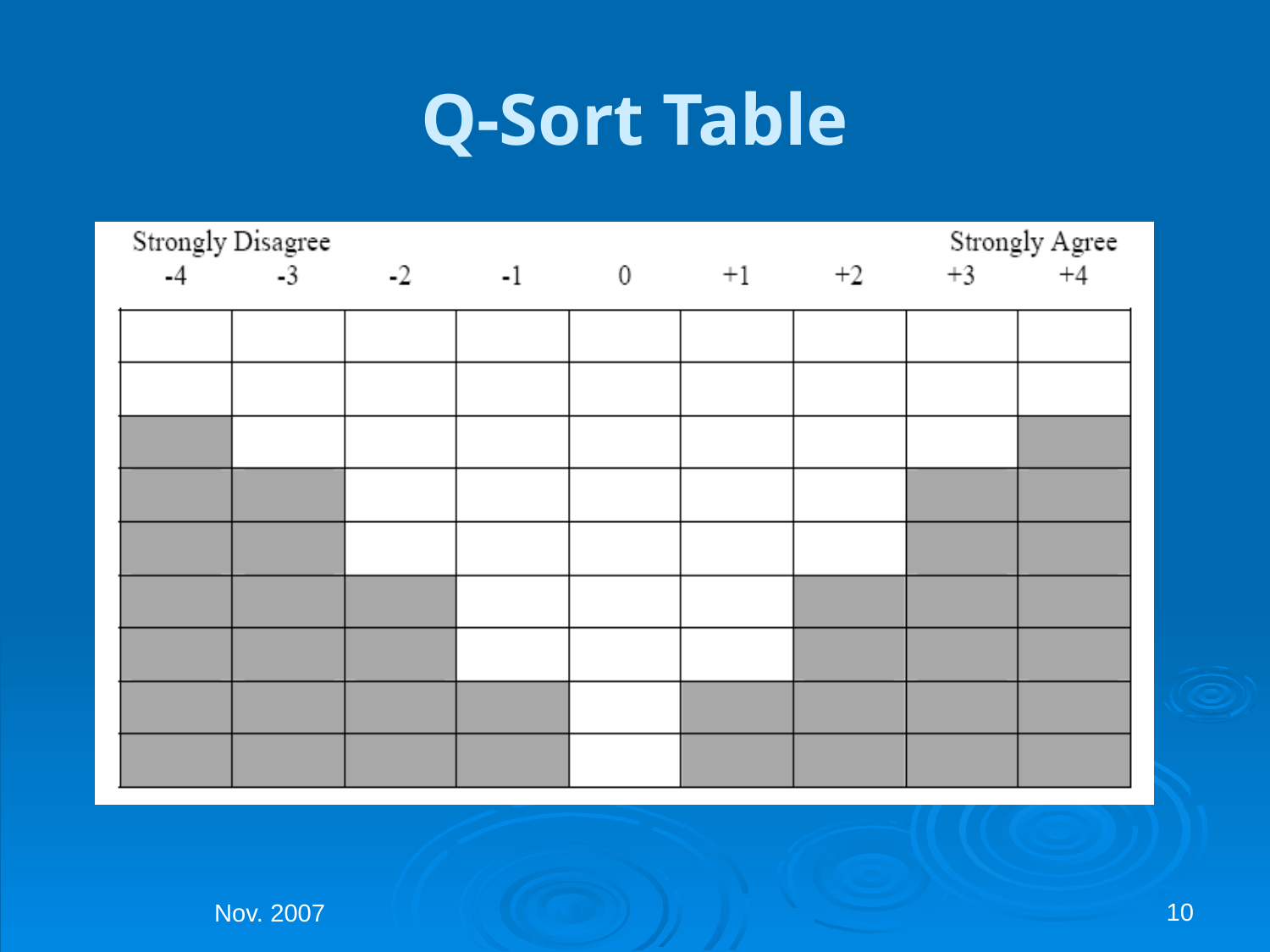

# Q-Sort Table
10
Nov. 2007

## Slide 11
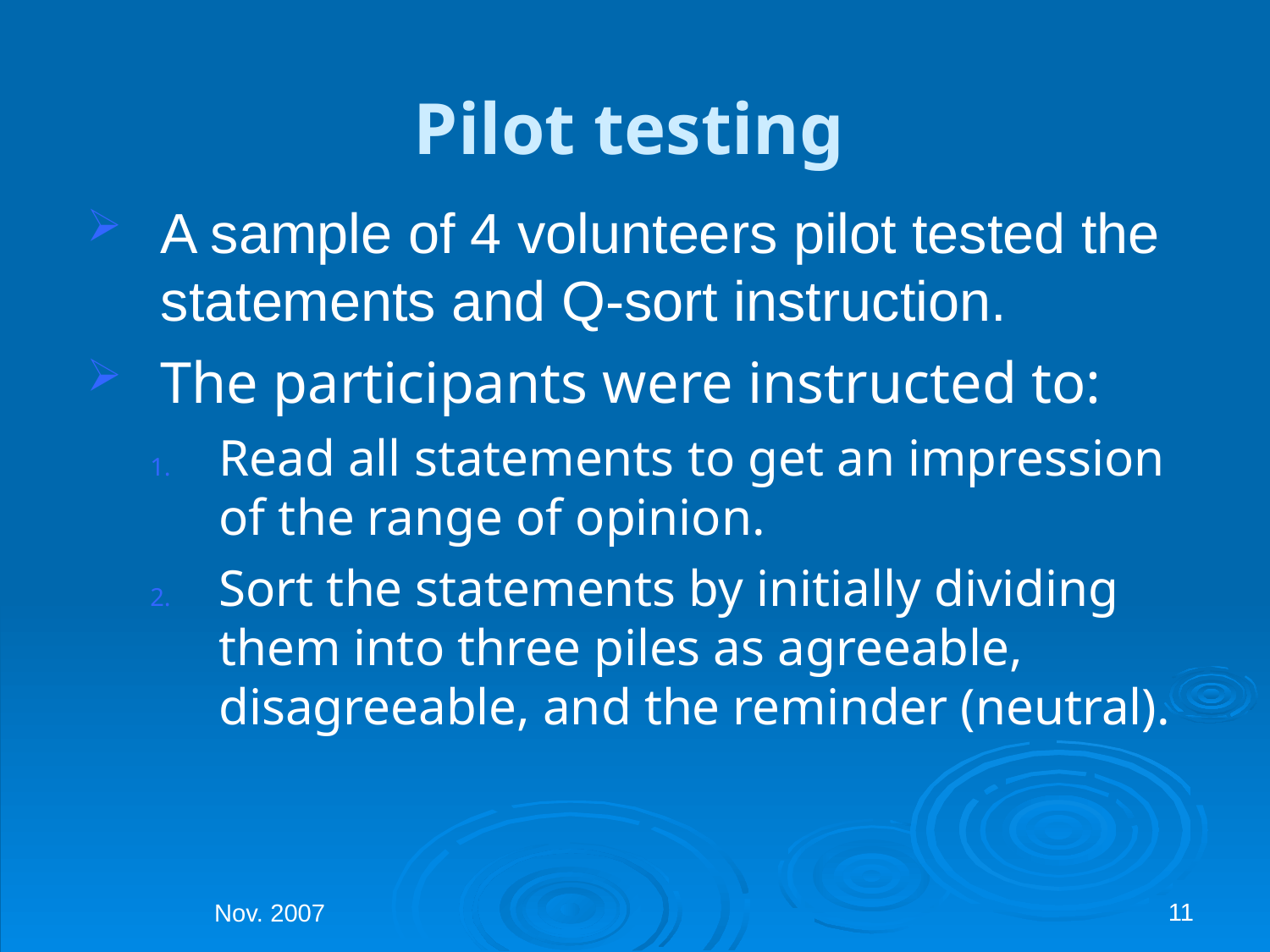

# Pilot testing
A sample of 4 volunteers pilot tested the statements and Q-sort instruction.
The participants were instructed to:
Read all statements to get an impression of the range of opinion.
Sort the statements by initially dividing them into three piles as agreeable, disagreeable, and the reminder (neutral).
11
Nov. 2007

## Slide 12
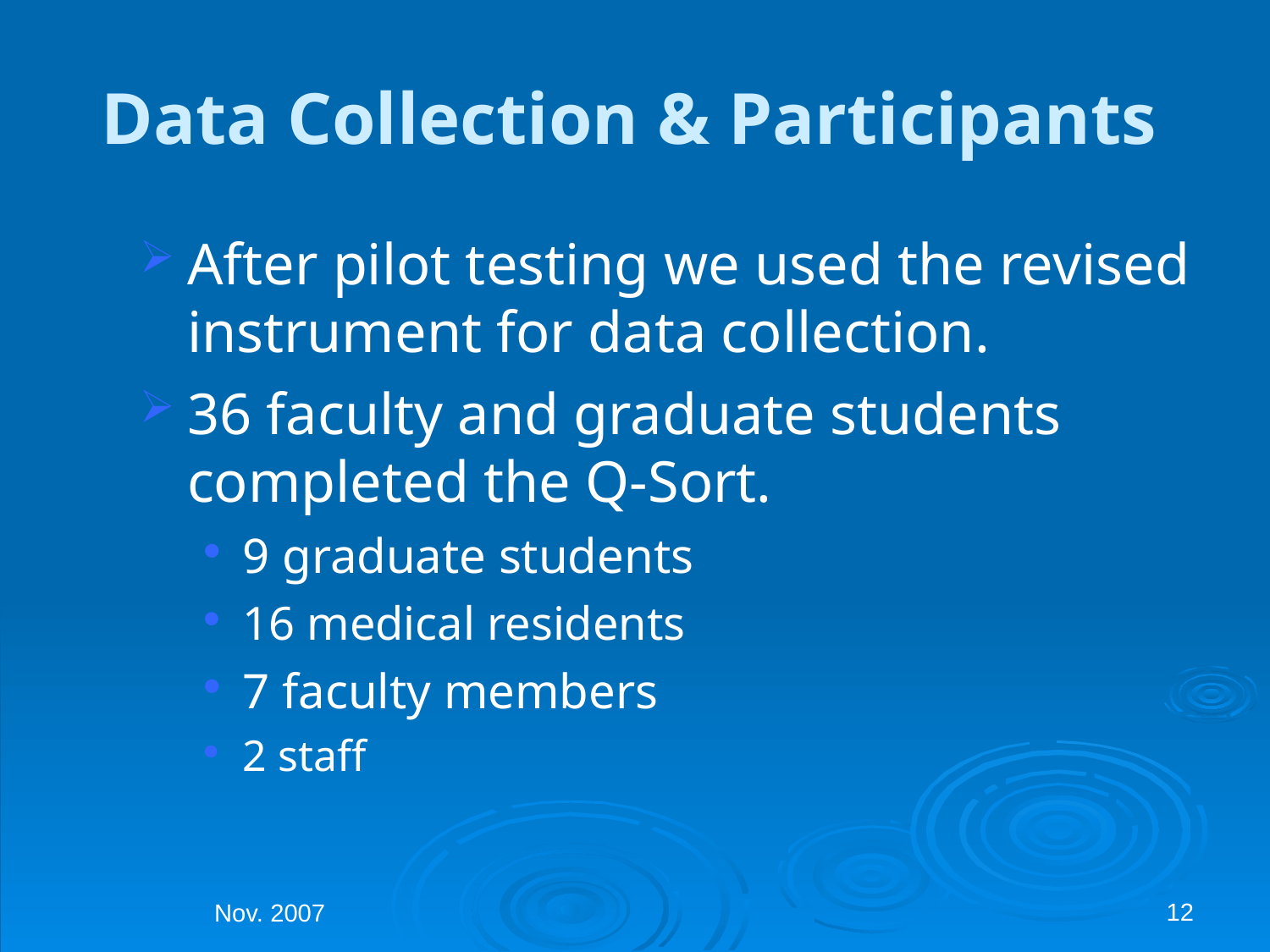

# Data Collection & Participants
After pilot testing we used the revised instrument for data collection.
36 faculty and graduate students completed the Q-Sort.
9 graduate students
16 medical residents
7 faculty members
2 staff
12
Nov. 2007

## Slide 13
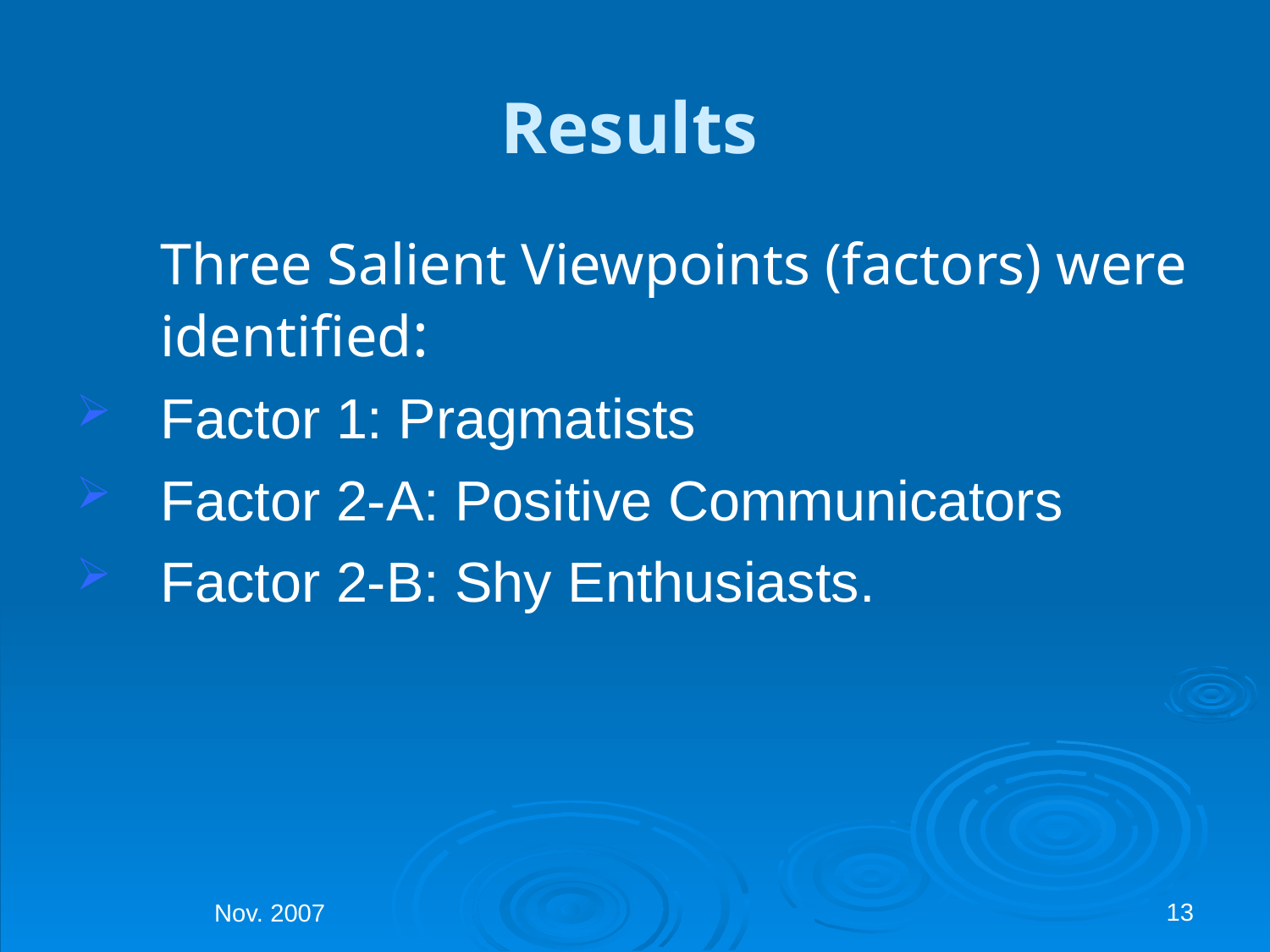

# Results
Three Salient Viewpoints (factors) were identified:
Factor 1: Pragmatists
Factor 2-A: Positive Communicators
Factor 2-B: Shy Enthusiasts.
13
Nov. 2007

## Slide 14
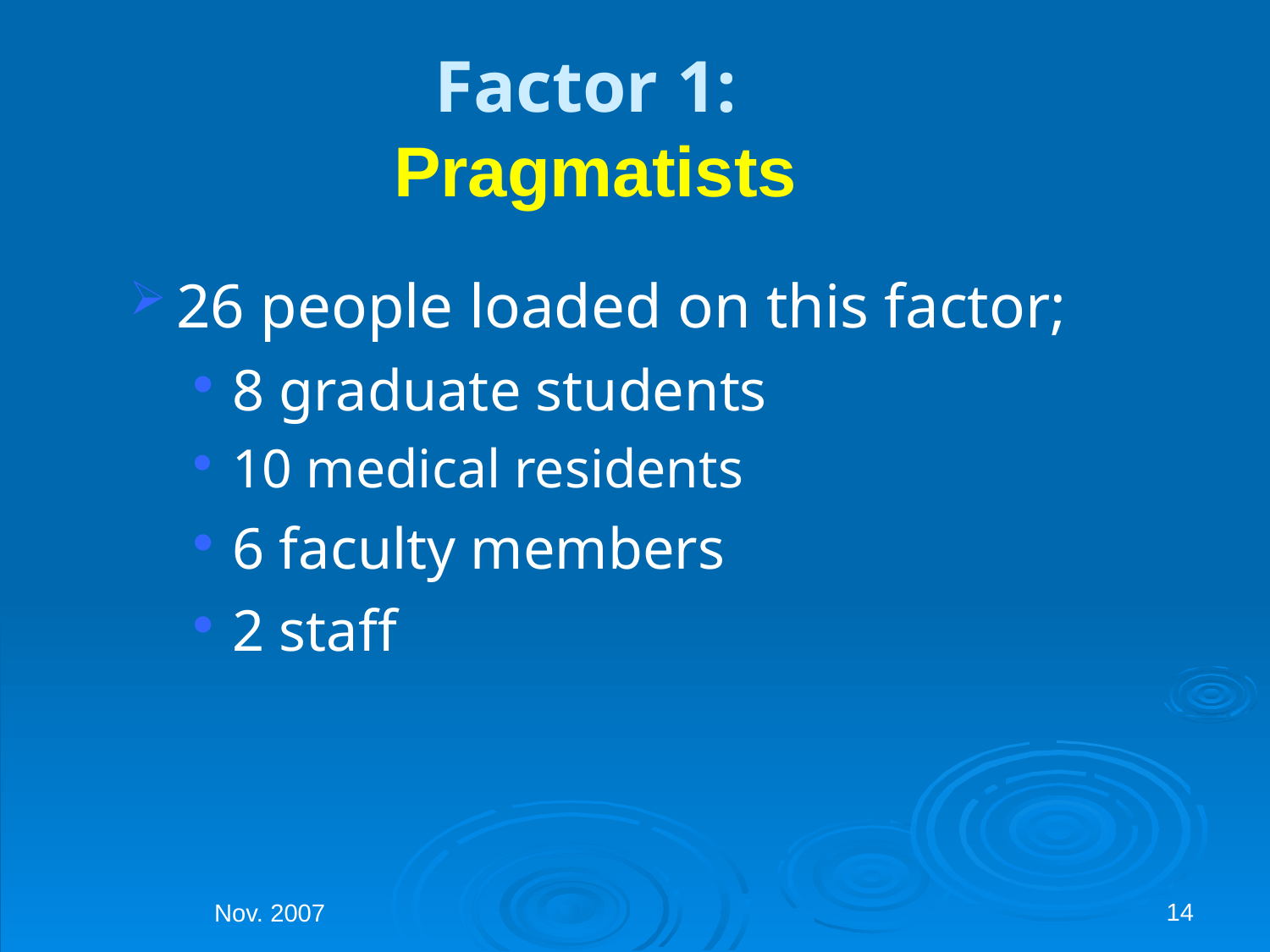

# Factor 1: Pragmatists
26 people loaded on this factor;
8 graduate students
10 medical residents
6 faculty members
2 staff
14
Nov. 2007

## Slide 15
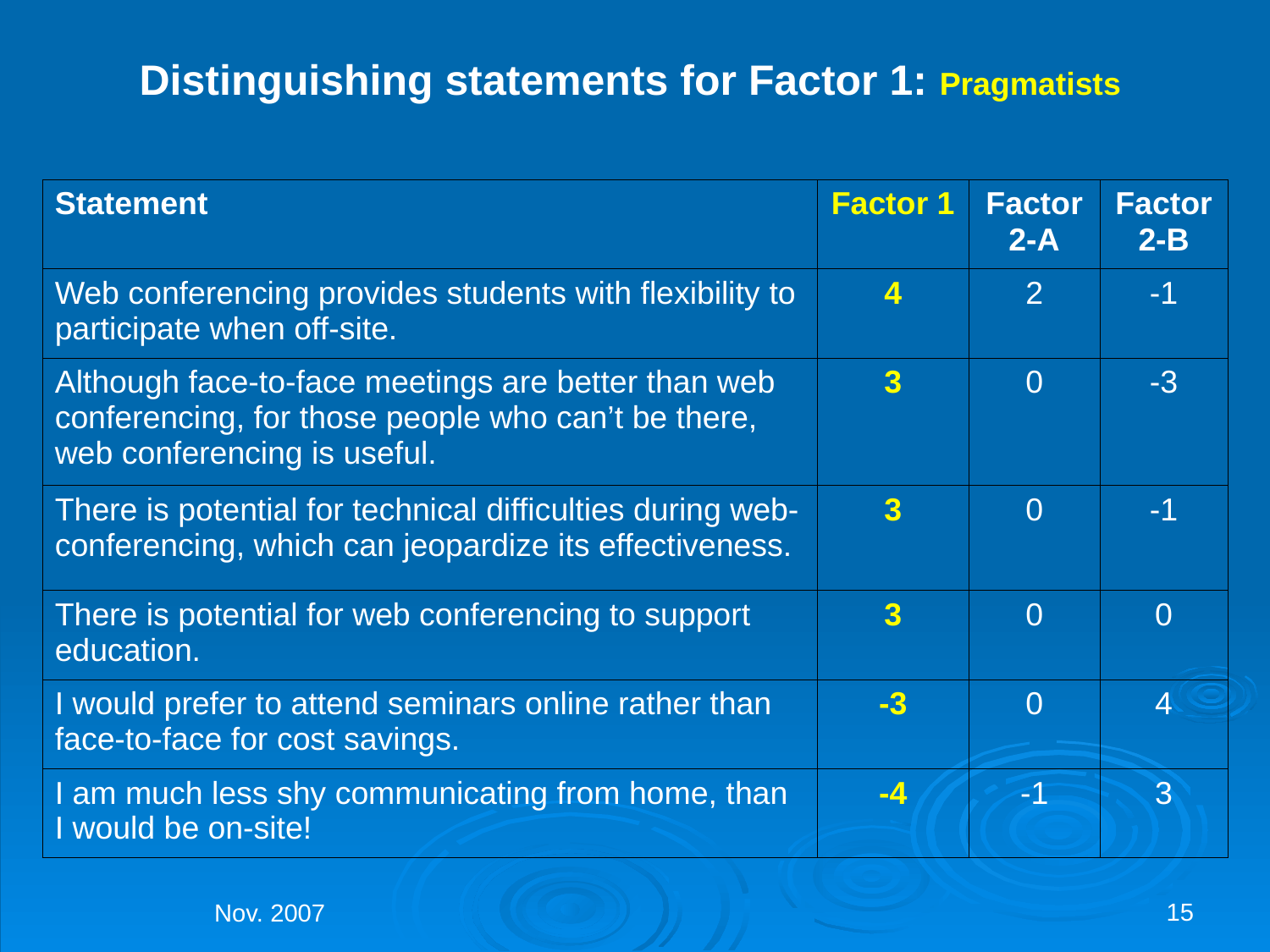

Distinguishing statements for Factor 1: Pragmatists
| Statement | Factor 1 | Factor 2-A | Factor 2-B |
| --- | --- | --- | --- |
| Web conferencing provides students with flexibility to participate when off-site. | 4 | 2 | -1 |
| Although face-to-face meetings are better than web conferencing, for those people who can’t be there, web conferencing is useful. | 3 | 0 | -3 |
| There is potential for technical difficulties during web-conferencing, which can jeopardize its effectiveness. | 3 | 0 | -1 |
| There is potential for web conferencing to support education. | 3 | 0 | 0 |
| I would prefer to attend seminars online rather than face-to-face for cost savings. | -3 | 0 | 4 |
| I am much less shy communicating from home, than I would be on-site! | -4 | -1 | 3 |
15
Nov. 2007

## Slide 16
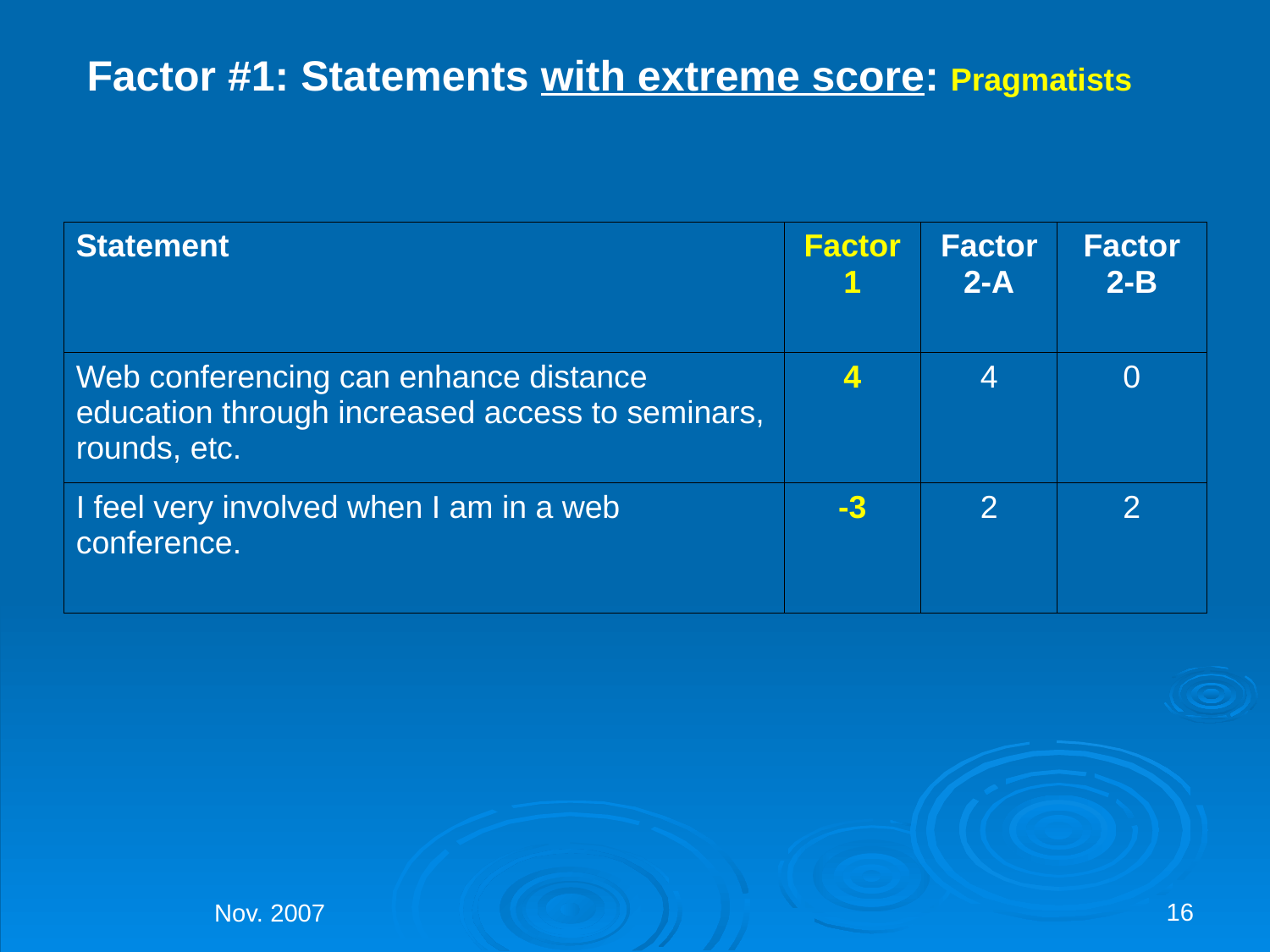

Factor #1: Statements with extreme score: Pragmatists
| Statement | Factor 1 | Factor 2-A | Factor 2-B |
| --- | --- | --- | --- |
| Web conferencing can enhance distance education through increased access to seminars, rounds, etc. | 4 | 4 | 0 |
| I feel very involved when I am in a web conference. | -3 | 2 | 2 |
16
Nov. 2007

## Slide 17
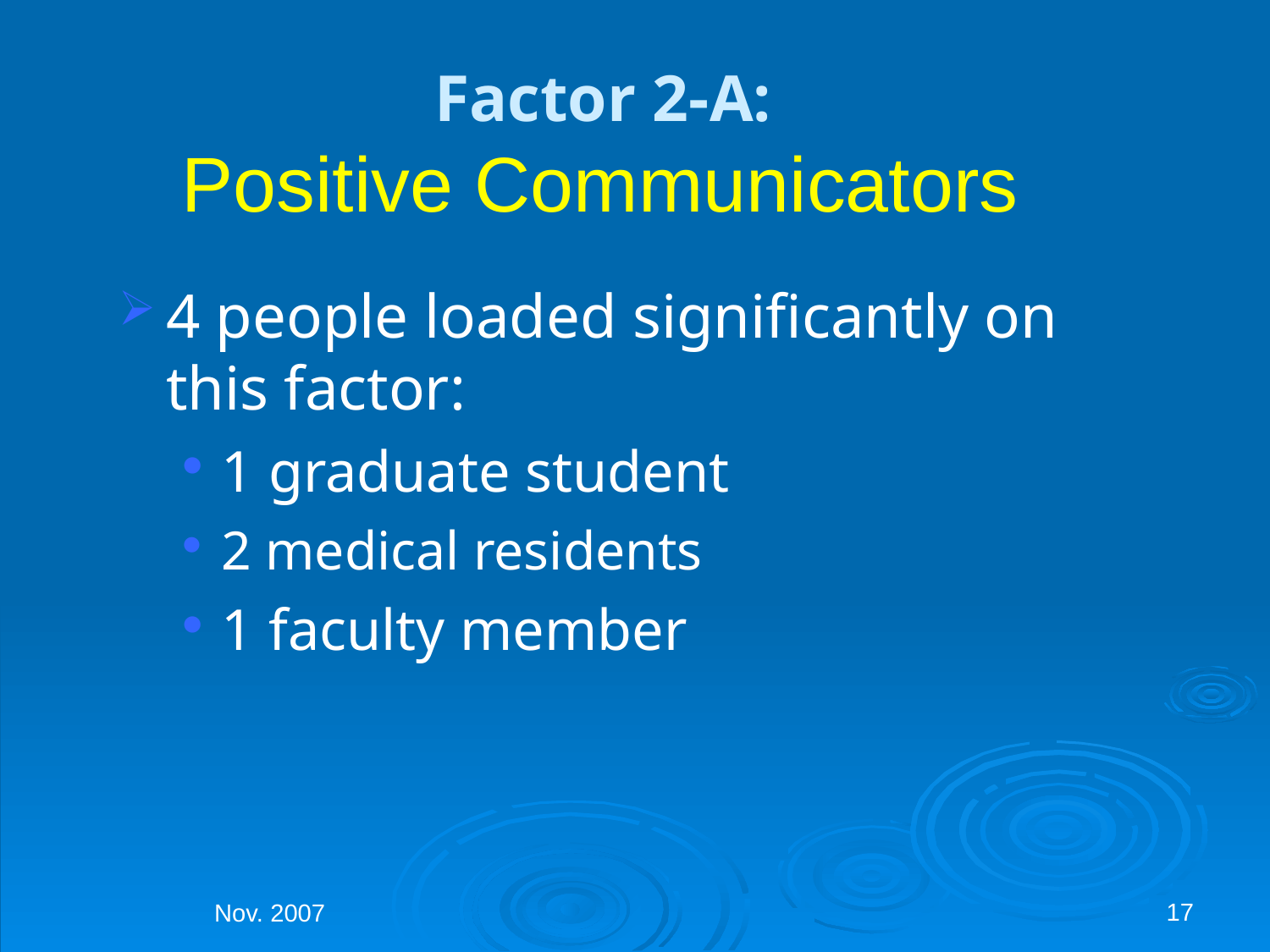

# Factor 2-A: Positive Communicators
4 people loaded significantly on this factor:
1 graduate student
2 medical residents
1 faculty member
17
Nov. 2007

## Slide 18
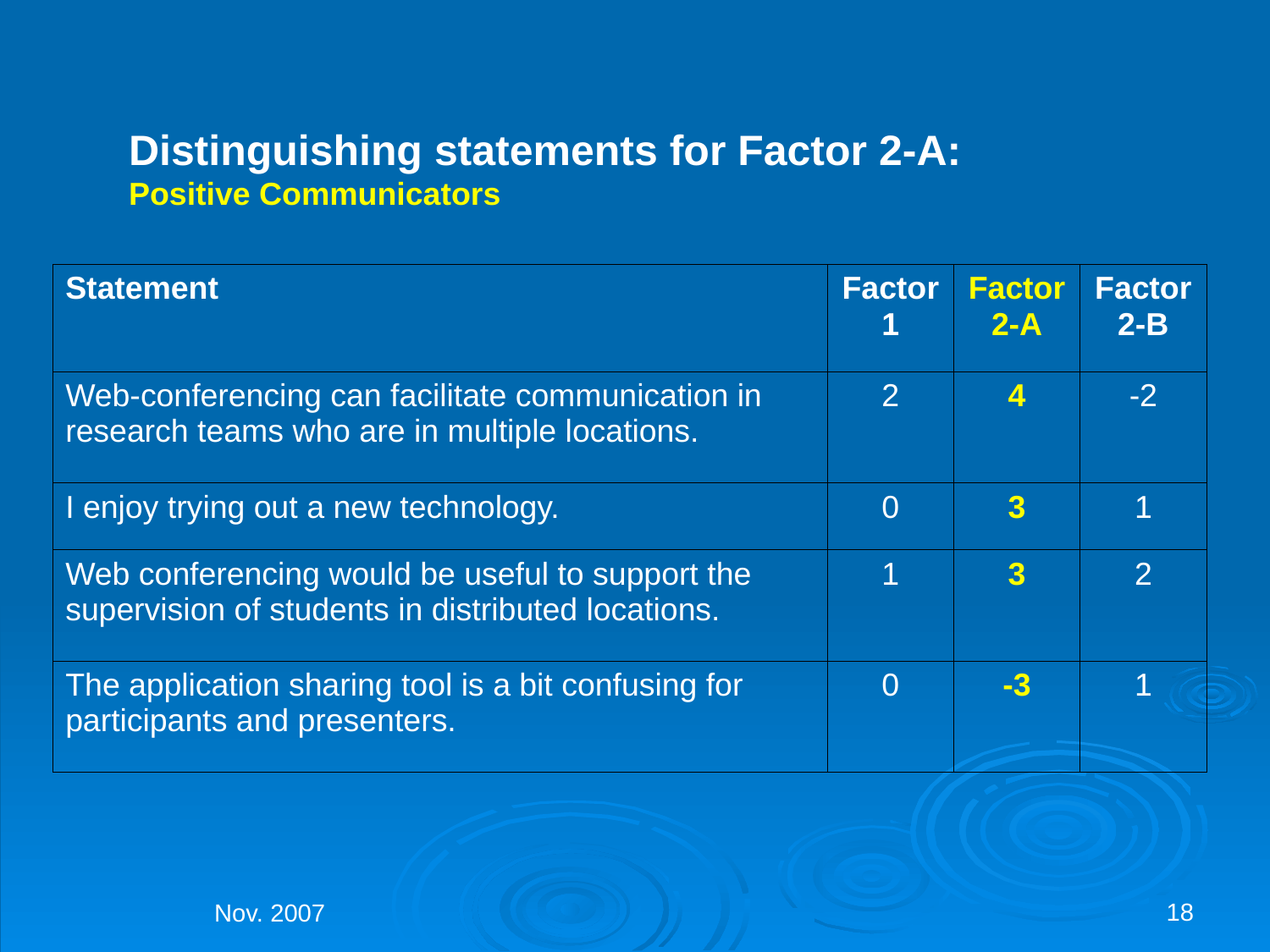

Distinguishing statements for Factor 2-A: Positive Communicators
| Statement | Factor 1 | Factor 2-A | Factor 2-B |
| --- | --- | --- | --- |
| Web-conferencing can facilitate communication in research teams who are in multiple locations. | 2 | 4 | -2 |
| I enjoy trying out a new technology. | 0 | 3 | 1 |
| Web conferencing would be useful to support the supervision of students in distributed locations. | 1 | 3 | 2 |
| The application sharing tool is a bit confusing for participants and presenters. | 0 | -3 | 1 |
18
Nov. 2007

## Slide 19
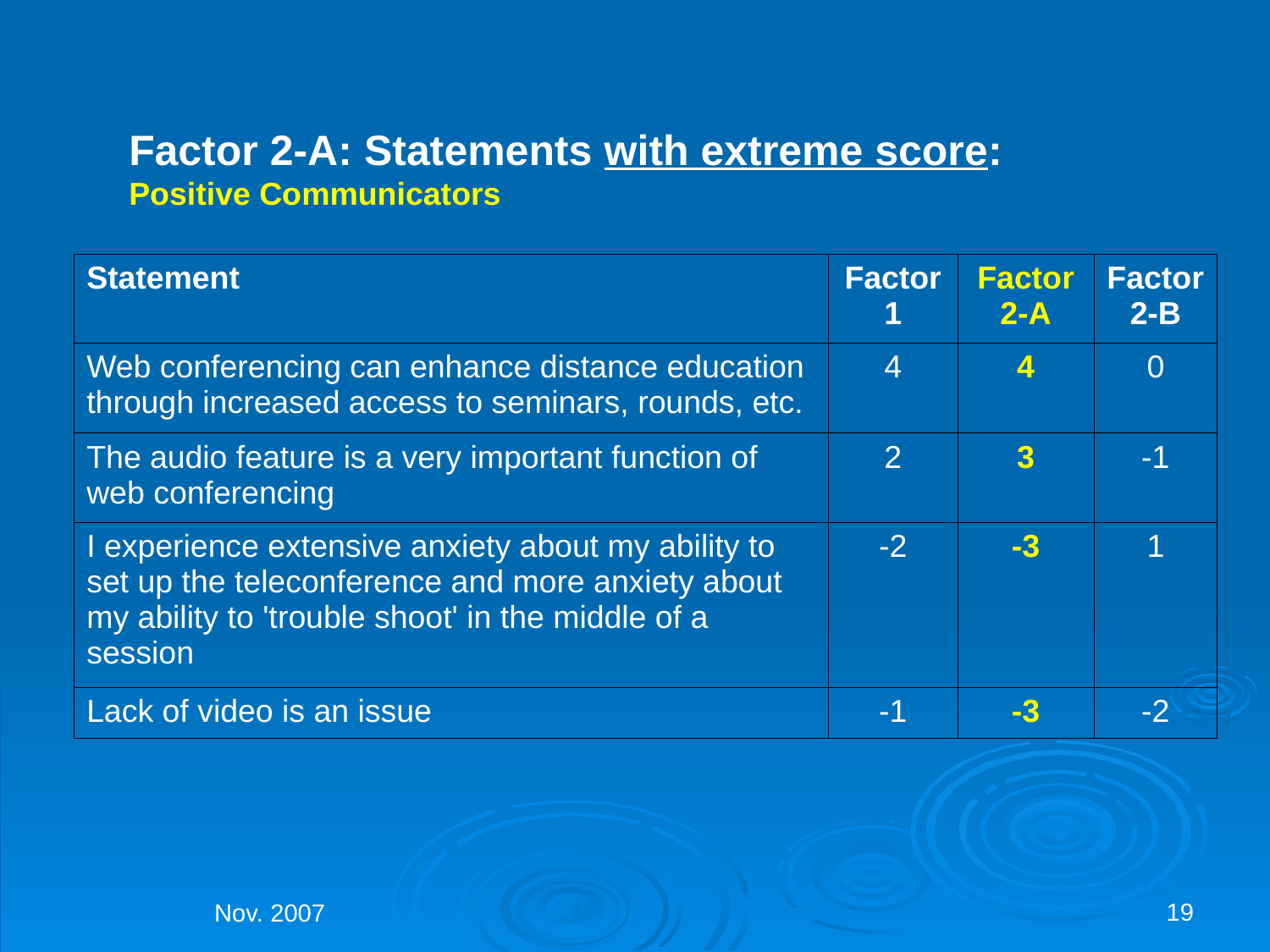

Factor 2-A: Statements with extreme score: Positive Communicators
| Statement | Factor 1 | Factor 2-A | Factor 2-B |
| --- | --- | --- | --- |
| Web conferencing can enhance distance education through increased access to seminars, rounds, etc. | 4 | 4 | 0 |
| The audio feature is a very important function of web conferencing | 2 | 3 | -1 |
| I experience extensive anxiety about my ability to set up the teleconference and more anxiety about my ability to 'trouble shoot' in the middle of a session | -2 | -3 | 1 |
| Lack of video is an issue | -1 | -3 | -2 |
19
Nov. 2007

## Slide 20
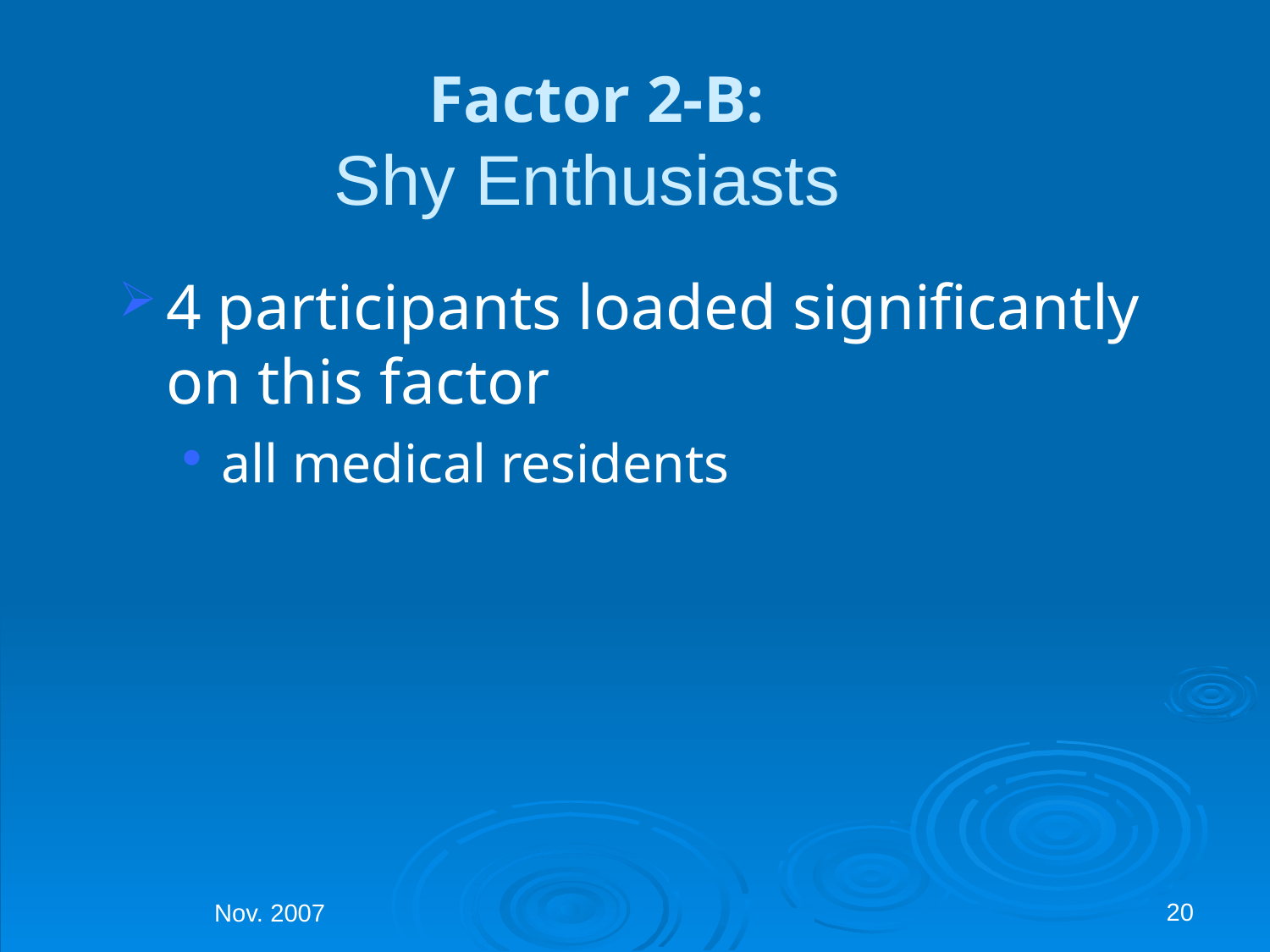

# Factor 2-B: Shy Enthusiasts
4 participants loaded significantly on this factor
all medical residents
20
Nov. 2007

## Slide 21
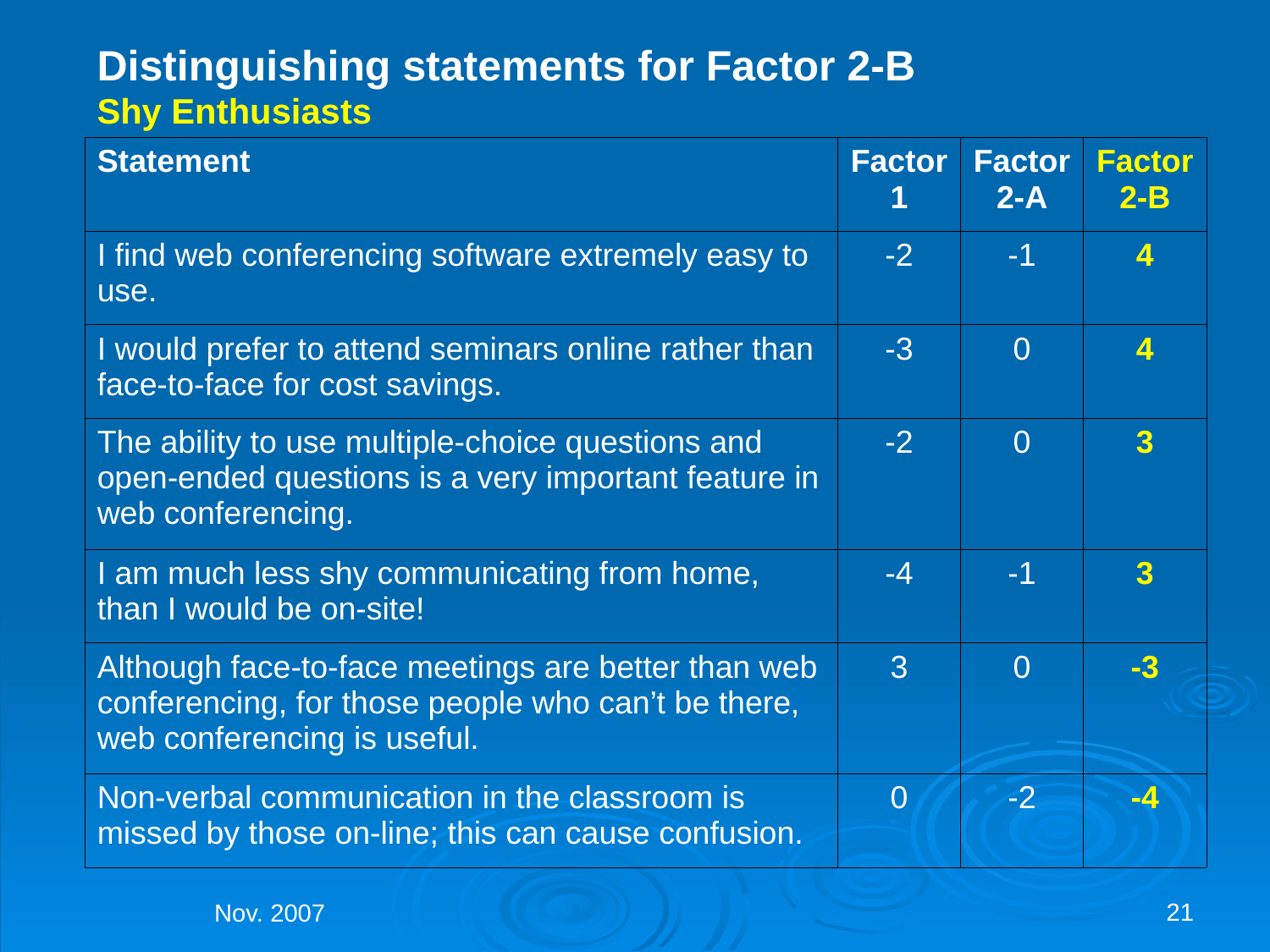

Distinguishing statements for Factor 2-B
Shy Enthusiasts
| Statement | Factor 1 | Factor 2-A | Factor 2-B |
| --- | --- | --- | --- |
| I find web conferencing software extremely easy to use. | -2 | -1 | 4 |
| I would prefer to attend seminars online rather than face-to-face for cost savings. | -3 | 0 | 4 |
| The ability to use multiple-choice questions and open-ended questions is a very important feature in web conferencing. | -2 | 0 | 3 |
| I am much less shy communicating from home, than I would be on-site! | -4 | -1 | 3 |
| Although face-to-face meetings are better than web conferencing, for those people who can’t be there, web conferencing is useful. | 3 | 0 | -3 |
| Non-verbal communication in the classroom is missed by those on-line; this can cause confusion. | 0 | -2 | -4 |
21
Nov. 2007

## Slide 22
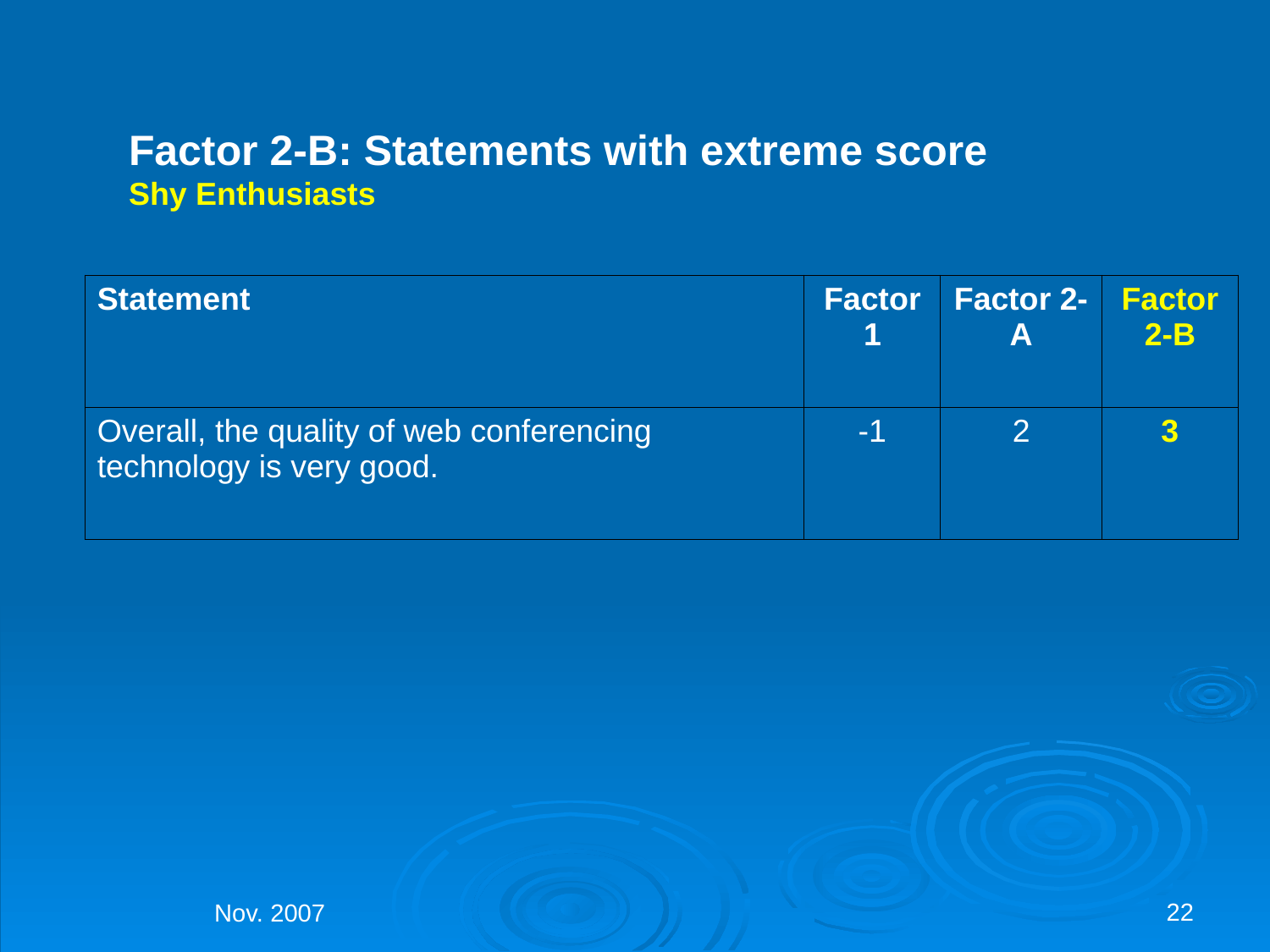

Factor 2-B: Statements with extreme score
Shy Enthusiasts
| Statement | Factor 1 | Factor 2-A | Factor 2-B |
| --- | --- | --- | --- |
| Overall, the quality of web conferencing technology is very good. | -1 | 2 | 3 |
22
Nov. 2007

## Slide 23
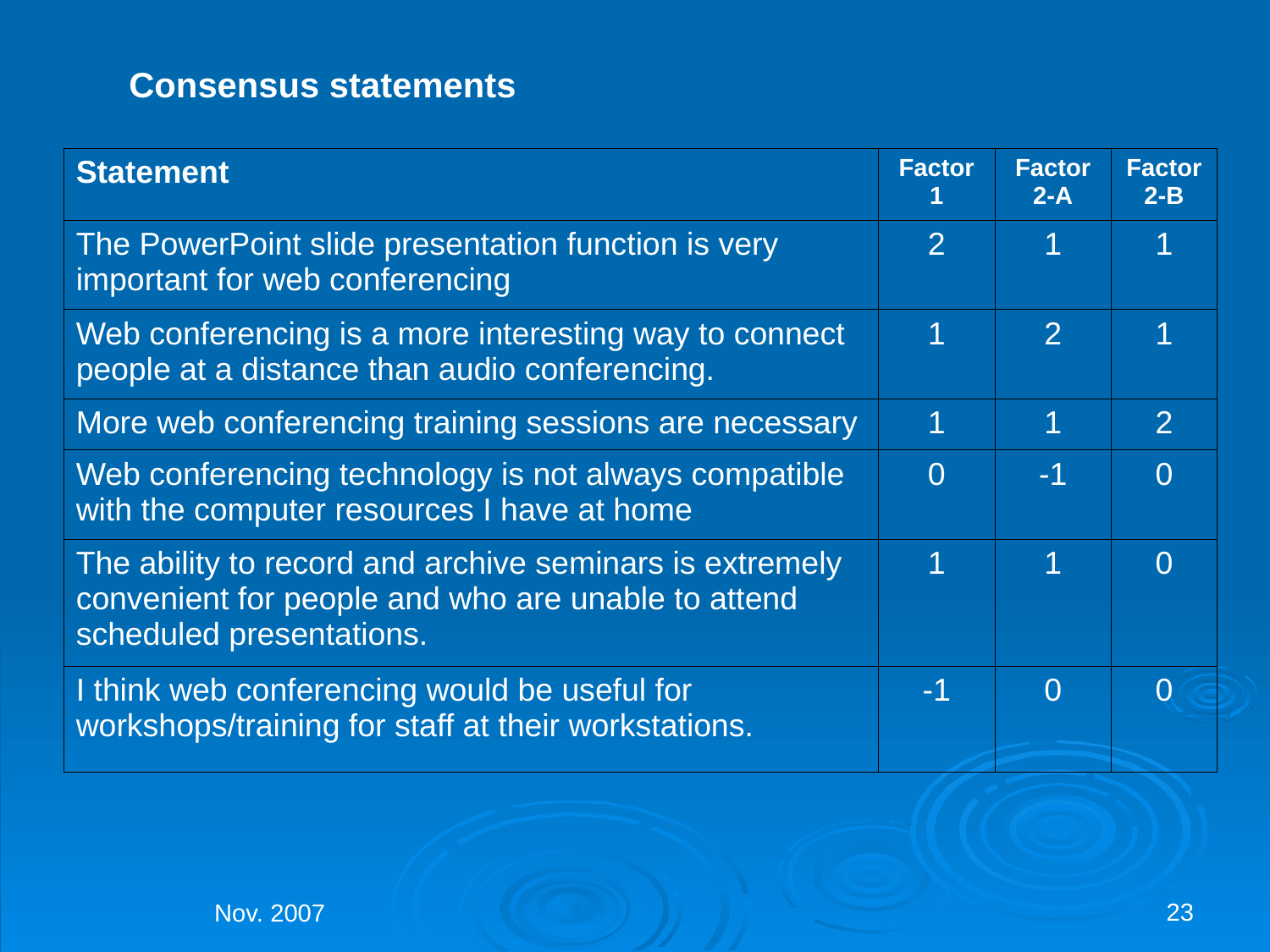

Consensus statements
| Statement | Factor 1 | Factor 2-A | Factor 2-B |
| --- | --- | --- | --- |
| The PowerPoint slide presentation function is very important for web conferencing | 2 | 1 | 1 |
| Web conferencing is a more interesting way to connect people at a distance than audio conferencing. | 1 | 2 | 1 |
| More web conferencing training sessions are necessary | 1 | 1 | 2 |
| Web conferencing technology is not always compatible with the computer resources I have at home | 0 | -1 | 0 |
| The ability to record and archive seminars is extremely convenient for people and who are unable to attend scheduled presentations. | 1 | 1 | 0 |
| I think web conferencing would be useful for workshops/training for staff at their workstations. | -1 | 0 | 0 |
23
Nov. 2007

## Slide 24
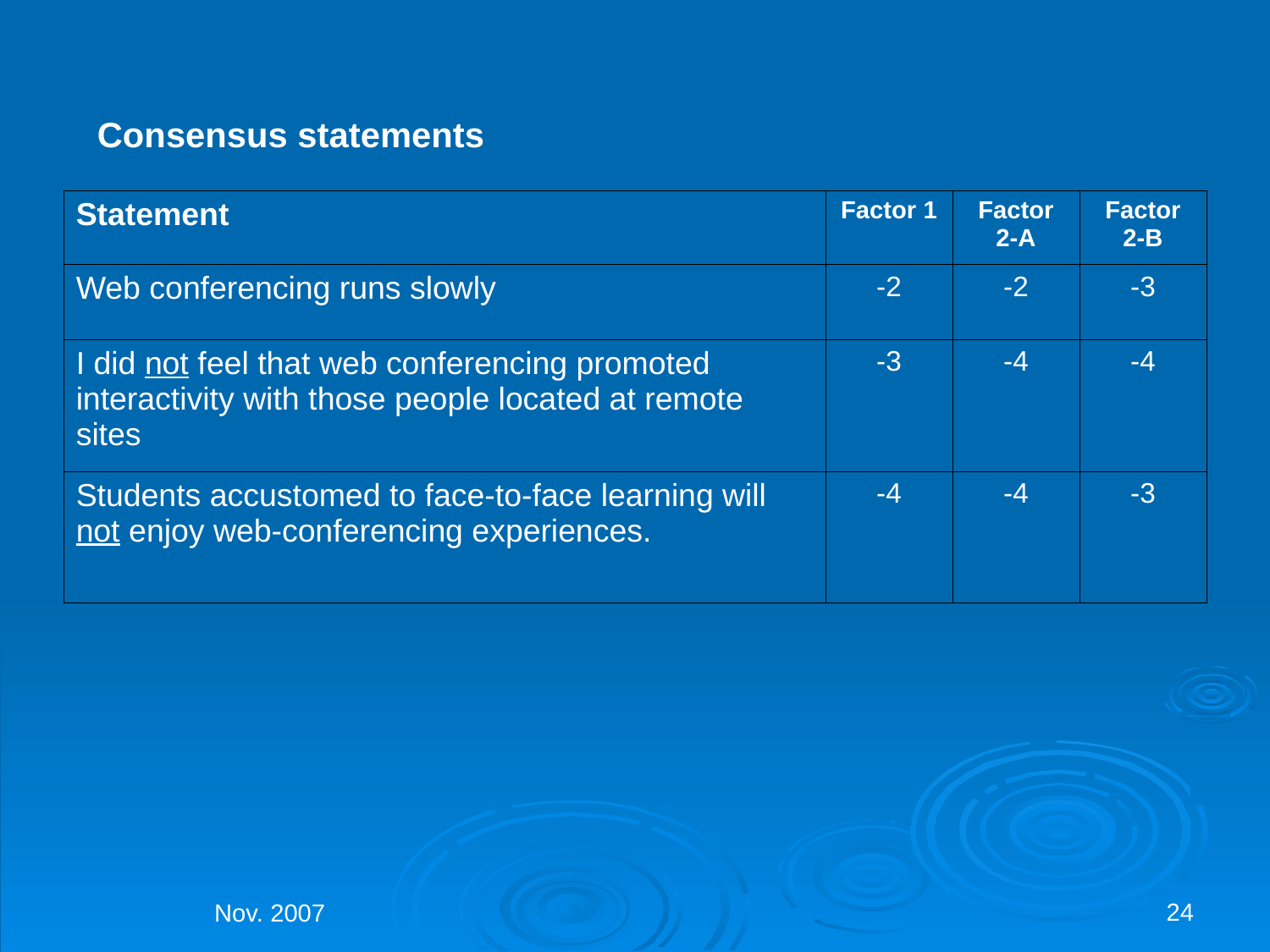

Consensus statements
| Statement | Factor 1 | Factor 2-A | Factor 2-B |
| --- | --- | --- | --- |
| Web conferencing runs slowly | -2 | -2 | -3 |
| I did not feel that web conferencing promoted interactivity with those people located at remote sites | -3 | -4 | -4 |
| Students accustomed to face-to-face learning will not enjoy web-conferencing experiences. | -4 | -4 | -3 |
24
Nov. 2007

## Slide 25
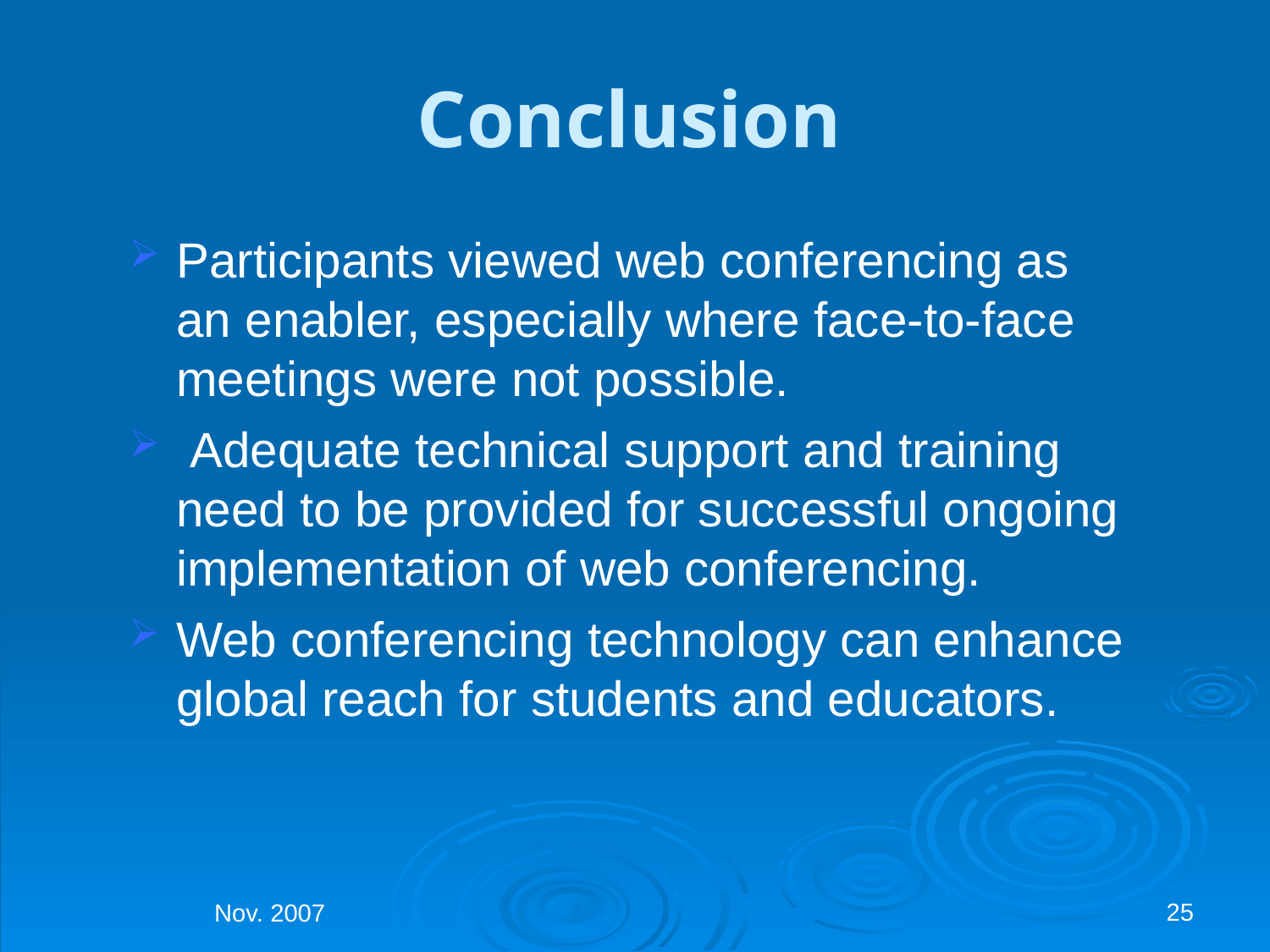

# Conclusion
Participants viewed web conferencing as an enabler, especially where face-to-face meetings were not possible.
 Adequate technical support and training need to be provided for successful ongoing implementation of web conferencing.
Web conferencing technology can enhance global reach for students and educators.
25
Nov. 2007
